# Identification of Estrogen Target Genes during Zebrafish Embryonic Development through Transcriptomic Analysis

**DOI:** 10.1371/journal.pone.0079020

**Published:** 2013-11-06

**Authors:** Ruixin Hao, Maria Bondesson, Amar V. Singh, Anne Riu, Catherine W. McCollum, Thomas B. Knudsen, Daniel A. Gorelick, Jan-Åke Gustafsson

**Affiliations:** 1 Center for Nuclear Receptors and Cell Signaling, Department of Biology and Biochemistry, University of Houston, Houston, Texas, United States of America; 2 National Center for Computational Toxicology, Office of Research and Development, U.S. Environmental Protection Agency, Research Triangle Park, North Carolina, United States of America; 3 Department of Embryology, Carnegie Institute for Science, Baltimore, Maryland, United States of America; 4 Department of Biosciences and Nutrition, Karolinska Institutet, Huddinge, Sweden; National University of Singapore, Singapore

## Abstract

Estrogen signaling is important for vertebrate embryonic development. Here we have used zebrafish (*Danio rerio*) as a vertebrate model to analyze estrogen signaling during development. Zebrafish embryos were exposed to 1 µM 17β-estradiol (E2) or vehicle from 3 hours to 4 days post fertilization (dpf), harvested at 1, 2, 3 and 4 dpf, and subjected to RNA extraction for transcriptome analysis using microarrays. Differentially expressed genes by E2-treatment were analyzed with hierarchical clustering followed by biological process and tissue enrichment analysis. Markedly distinct sets of genes were up and down-regulated by E2 at the four different time points. Among these genes, only the well-known estrogenic marker *vtg1* was co-regulated at all time points. Despite this, the biological functional categories targeted by E2 were relatively similar throughout zebrafish development. According to knowledge-based tissue enrichment, estrogen responsive genes were clustered mainly in the liver, pancreas and brain. This was in line with the developmental dynamics of estrogen-target tissues that were visualized using transgenic zebrafish containing estrogen responsive elements driving the expression of GFP (*Tg*(*5xERE:GFP*)). Finally, the identified embryonic estrogen-responsive genes were compared to already published estrogen-responsive genes identified in male adult zebrafish (Gene Expression Omnibus database). The expressions of a few genes were co-regulated by E2 in both embryonic and adult zebrafish. These could potentially be used as estrogenic biomarkers for exposure to estrogens or estrogenic endocrine disruptors in zebrafish. In conclusion, our data suggests that estrogen effects on early embryonic zebrafish development are stage- and tissue- specific.

## Introduction

Estrogen signaling through its main components cytochrome P450 aromatase (CYP19) and the estrogen receptors (ERs), is well conserved through the evolution of vertebrates (reviewed in 1). This conservation implies important roles for estrogenic pathways in a host of tissues and sexually dimorphic organs including the reproductive tract, brain, liver, heart, breast, skin and bone. As a consequence, excess or deficiency of estrogen can lead to pathological conditions such as infertility, cancer, and osteoporosis. In addition to having important functions in the adult, estrogens are crucial for normal embryonic development, manifested by perturbed development of brain and gonads as well as aberrant behavior in both aromatase and ER knockout mice [2,3].

The genome of the vertebrate zebrafish (*Danio rerio*) codes for three estrogen receptors, Esr1, Esr2a and Esr2b (previously denoted ERα, ERβ2 and ERβ1, respectively) [4]. These receptors presumably mediate the genomic responses to estrogen signaling through their function as DNA-binding transcription factors. A fourth estrogen targeted receptor, the membrane localized G protein-coupled estrogen receptor 1 (Gper), induces activation of the so called non-genomic response, including phosphorylation of the mitogen-activated protein kinases MAPK3/MAPK1 [5], which eventually also results in downstream transcriptional changes.

The fetal expression of the different estrogen receptors during zebrafish development is induced at 24 hours post fertilization (hpf) [4]. The most highly expressed ER in early development has been suggested to be Esr2a. The *esr2a* mRNA is maternally loaded to the oocyte, disappears between 6 and 12 hpf and returns with the start of the zygotic expression after 1 day post fertilization (dpf) [4,6]. *In situ* hybridization of *esr2a* shows that its expression is high in the head/brain region and in proximity to the yolk at 24 to 48 hpf [7,8]. *esr2a* mRNA is also expressed during early life stage in the epidermis, pectoral ﬁn buds, hatching gland and is distinctly expressed in neuromast cells of both the anterior and the posterior lateral line [7,9]. Although the expression of estrogen receptors has been profiled during embryonic zebrafish development, knowledge of estrogen signaling at early developmental stages is limited. 

It is reasonable to surmise that estrogen activity is important for the development of the tissues and organs in which the estrogen receptors are expressed. Consequently, knock down of estrogen receptor expression, or treatment with excess levels of agonists or antagonists would be expected to perturb development of these tissues and organs. In support of this hypothesis, morpholino knock down of *esr2a* efficiently decreases the formation of neuromasts, showing a direct role for *esr2a* in their development [9]. The most studied effect of excess estrogen or xenoestrogen exposure of zebrafish is the change in sex ratio and fertility, decreasing both the percentage of males and their fertility ([10] and references therein). On the contrary, treatment of zebrafish during 48-168 hpf with an aromatase inhibitor, which induces estrogen deficiency, causes neurobehavioral deficits, including altered tactile response, swimming movements, vestibular behavior, and pectoral fin and eye movements [11]. After prolonged treatment the fish die by cardiac arrest. These phenotypes can be rescued by a simultaneous addition of estrogen [11], implicating functional links to estrogen pathways. Estrogen deficiency also significantly diminishes thickness in most retinal layers, suggesting that estrogen is important for normal eye development [12]. Thus, consistency emerges when comparing the tissues affected by exposure to or inhibition of estrogen to the tissues that have ER expression. 

Several biomarkers of estrogenic exposure have been identified in zebrafish, including the liver-produced yolk proteins Vitellogenin 1 and 3 (encoded by *vtg1 and vtg3*), and the brain-specific Aromatase B (AroB, encoded by *cyp19a1b*). These markers have been used to detect estrogenic endocrine disrupters both in laboratory raised zebrafish and in field monitoring of other fish species. To identify additional E2-target genes, studies have been performed in zebrafish using a large-scale transcriptomic approach. In one study, total mRNA from adult male zebrafish was analyzed on a custom-made microarray set containing about 16 K oligonucleotide probes [13]. Approximately 1,000 estrogen-responsive genes were identified, including the already known target genes *vtg1*, *vtg3* and *esr1*. Three other studies analyzed gene expression changes in liver of adult male zebrafish after E2 treatment using 14-16K microarray platforms [14-16], and identified hepatic E2-responsive genes. While the first study describes that the estrogen target genes are highly represented among cell proliferation, apoptosis and gene expression functional categories, the other studies report that estrogen target genes are involved in metabolism. This observation logically reflects liver function [13-16]. Together, these studies identify a number of previously unknown estrogen target genes in the entire adult male organism or livers of zebrafish that potentially could serve as new biomarkers; however, a bioinformatic comparison of the genes described in the different publications has not yet been performed. 

In this study, we describe a whole-genome analysis of estrogen regulated genes in zebrafish embryos at four early developmental stages. We used an Agilent zebrafish gene expression microarray with 44K probes to analyze organism wide expression changes induced by E2, and applied biological functional process and tissue enrichment analysis to interpret the consequences of these changes for early embryonic development. We further compared the embryonic E2-target genes to previously published estrogen-responsive genes in male adult zebrafish to identify potential biomarkers that could be used to detect xenoestrogenic exposures both to embryos and adult zebrafish. 

## Materials and Methods

### Zebrafish maintenance

The zebrafish work was conducted according to relevant national and international guidelines. Wild-type strains DZ and TAB14, as well as transgenic fish lines *Tg*(*5×ERE:GFP*) [17] and *Tg*(*ins:mCherry*) were used according to the maintenance and experimental protocols approved by the Institutional Animal Care and Use Committee at University of Houston (protocol numbers protocol numbers 12-042 and 10-040 approved at Sept. 17, 2012 and Nov. 19 2012, respectively). Adult zebrafish were maintained in 2.5 Liter polyethylene tanks in a Z-MODE holding system from Aquatic Habitat, (Aquatic Habitats Inc., Apopka, FL) or 3.5 liter tanks in a Tecniplast system (Tecniplast USA Inc., West Chester, PA) supplied continuously with circulating filtered water at 28°C under 14 h of light and 10 h of dark cycle (14:10 LD; lights on 8 AM; lights off 10 PM.). The fish were fed commercial flake food (Aquatic Habitat) in the morning, baby brine shrimp (Brine Shrimp Direct, Ogden, UT) at noon and Cyclop-eeze (Argent Chemical Laboratory, Redmond, WA) in the evening during the week. On weekends they were fed baby brine shrimp and Cyclop-eeze.

### Embryo and adult zebrafish treatments

After breeding adult fish from the different fish lines, embryos were collected and allowed to develop in a Petri dish at 28.5°C. 17β-estradiol (E2) (Sigma-Aldrich, St. Louis, MO) at a 1 mM stock solution in 100% dimethylsulfoxide (DMSO) was diluted in embryo medium (5 mM NaCl, 0.17 mM KCl, 0.33 mM CaCl_2_, 0.33 mM MgSO_4_) to obtain 1 μM concentrations. Clutches of zebrafish embryos from several pairs of adult fish were divided and transferred into 6 well plates. 30 embryos were pooled as one biological sample and exposed to 3 ml 1 μM E2 or vehicle (0.1% DMSO) from approximately 3 hpf. Embryo media containing E2 or DMSO were renewed every day. At different time points, 1 dpf (24 hpf), 2 dpf (48 hpf), 3 dpf (72 hpf) and approximately 4 dpf (4.3 dpf, 104 hpf), embryos were collected for analysis. 

For analysis of adult fish, 5 six months old male adult DZ fish/group were exposed in 500 mL fish water containing 1 μM E2 or vehicle DMSO (0.1%) and were maintained at 28°C for 48 h. After the treatment, fish were anesthetized and snap frozen in liquid nitrogen followed by RNA extraction. 

### RNA extraction and cDNA synthesis

Total RNA from pooled DZ embryos was extracted using Trizol (Invitrogen Corporation, Carlsbad, CA) and RNeasy spin columns (Qiagen, Chatsworth, CA) according to the manufacturer’s protocols. DNase I (Qiagen, Chatsworth, CA) digestion was performed to remove remaining DNA. RNA concentrations were measured with NanoDrop 1000 spectrophotometer (Agilent Technologies, Palo Alto, CA) and RNA integrity was analyzed with Agilent 2100 Bioanalyzer (Agilent Technologies, Palo Alto, CA). cDNA synthesis was carried out using Superscript II reverse transcriptase (Invitrogen Corporation, Carlsbad, CA). 

Frozen adult fish from E2 treatment and vehicle control (0.1% DMSO) groups were ground to a crude powder using pre-cooled mortars and pestles. Liquid nitrogen was added into the mortars to keep the samples frozen. Crude tissue powder was then transferred to pre-cooled 5 ml sterile centrifuge tubes (VWR, Houston, TX) containing Trizol (Invitrogen Corporation, Carlsbad, CA). Motorized homogenizer (Kinematica Polytron PT 1200 E, Lucerne, Switzerland) was used to completely homogenize the fish samples. Total RNA was extracted according to the procedures described above. 

### Microarray

Agilent zebrafish gene expression microarray v2 (part number G2519F and AMADID (design) 019161) was used for the microarray analysis. Experiments were performed at the Genomic and RNA Profiling Core (Baylor College of Medicine, Houston, TX). The Genomic and RNA Profiling Core first conducted Sample Quality checks using the Nanodrop ND-1000 and Agilent Bioanalyzer Nano chips. For labeling, the Agilent Quick Amp Labeling Kit (for one-color) Protocol Version 6.5 was used. 50ng of total RNA that had passed the quality check was used for the protocol as recommended by Agilent. The Labeling Kit (Agilent p/n 5190-0442) was used along with Agilent’s RNA Spike-In Kit, Agilent’s Hybridization Kit, and Agilent’s Wash Buffers 1 and 2. The RNA Spike-Ins was added to the sample. The sample was simultaneously amplified and Cy3 dye labeled as cRNA was generated using T7 RNA Polymerase. The cRNA was purified using Qiagen RNeasy mini spin columns. Samples were then measured again on the Nanodrop for yield and dye incorporation. The samples were then fragmented and 1.65 µg of sample and hybridization mix was loaded onto each of the 4x44K Expression arrays. The slide was hybridized in Agilent Hybridization Chamber at 65°C at a 10rpm rotation for 17 h. The slide was washed using the Agilent Expression Wash Buffer Set 1 and 2 as per the Agilent protocol. Once dry, the slides were scanned with the Agilent Scanner (G2565BA) using Scanner Version C and Agilent Feature Extraction Software Version 11.0.1.1. Time points 1 and 2 dpf were performed in biological triplicates of independent pools of RNA while time points 3 and 4 dpf were performed in quadruplicates. All biological replicates were prepared from different batches of embryos spawning from different breeding pairs, but from the same fish strain. The microarray results were submitted to Gene Expression Omnibus database (GSE42766).

### Real-time PCR

Real-time polymerase chain reaction (PCR) was performed using a 7500 Fast Real-Time PCR (Applied Biosystems, Foster City, CA) with Fast SYBR Green Master mix (Applied Biosystems, Foster City, CA). Primer BLAST (http://www.ncbi.nlm.nih.gov/tools/primer-blast/) was used to design the primers, which were synthesized by Integrated DNA Technologies, Inc (San Diego, CA). Primers of 18-22 base pairs (bp) were designed to amplify sequences of 100-300 bp (Table S1). Relative gene expression data was normalized against 18S ribosomal RNA (18S rRNA) expression and analyzed with unpaired two-tailed t-test. Each experiment was carried out at least three times with three technical repeats each time. Significance is presented at *P*≤0.05 (*) or *P*≤0.01 (**).

### Imaging

E2 treated *Tg*(*5×ERE:GFP*) embryos were used to follow estrogen responsive tissue development. Hybrid embryos obtained from *Tg*(*5xERE:GFP*) crossed with *Tg*(*ins:mCherry*) transgenic fish were used to track endocrine pancreas development. Embryo treatments were as described above. Fluorescence in the live embryos was visualized using a Nikon AZ100M microscope equipped with Nikon DS digital camera head and the NIS Elements imaging software (Nikon Instruments Inc, Melville, NY). To inhibit pigment formation, embryos and larvae were incubated in 200 μM 1-phenyl-2-thiourea (Sigma-Aldrich, St. Louis, MO) from 1 dpf. For live imaging, embryos and larvae were anesthetized with 0.04% MS-222 (Sigma-Aldrich, St. Louis, MO), mounted in 3% methylcellulose on a glass slide and imaged using a 4X objective. Fluorescent images were pseudo-colored, superimposed and adjusted using Adobe Photoshop CS5 (Adobe Systems Inc. Sam Jose, CA). 

### Quantification of E2 uptake

Wild type TAB14 embryos were treated with embryo media containing 1 µM E2 or vehicle (0.1% DMSO) using the protocol described above. Embryo media was collected after treatments and E2 levels remaining in the media were analyzed using HPLC (n=3). HPLC analyses were performed on a Binary HPLC pump 1525 (Waters, Milford, MA, USA) equipped with an autosampler 2707 injector and a photodiode array detector (PDA) 2998 set at 280nm (Waters). The HPLC system was based on a Nucleodur C18 column (250 x 4mm, 5µm, Macherey Nagel, Bethlehem, PA, USA) in the following conditions: mobile phases: A: 20 mM ammonium acetate pH 3.5/acetonitrile (95/5, v/v), B: 100% acetonitrile; Gradient: 0–2 min, A: 100% isocratic; 2-3 min, linear gradient from A: 100% to A:B 60:40; 3–8 min, A:B 60:40 isocratic; 8–9 min, linear gradient from A:B 60:40 to A:B: 50:50; 9-14 min, A:B 50:50 isocratic; 14–15 min, linear gradient from A:B 50:50 to B 100%; 15–19 min, B: 100% isocratic. In our system, E2 was eluted at a retention time of 11.5 min, and it was quantified by measuring the area under the peak (based on a standard curve previously established). 

### Whole-mount *in situ* hybridization (ISH)

All procedures of whole-mount *in situ* hybridization were performed as described previously [18]. Partial-length *vtg4* (630 bp) was amplified by PCR (95 °C for 10 min, 95 °C for 30 s, 50 °C for 30 s, 72 °C for 40 s (40 cycles), 72 °C for 5 min) from cDNA that was prepared from total RNA extracted from 1-month old adult male zebrafish treated with E2 (as described above) using primers forward 5’-GATCAATTAACCCTCACTAAAGGCCTATCATCGCCCGTGCTGTT-3’ and reverse 5’- GATCTAATACGACTCACTATAGGACAGTTCTGCATCAACACATCT-3’ for sense and antisense probes. These primers were designed to contain the T3 (forward primer) and T7 (reverse primer) promoter regions for sense and antisense transcripts, respectively. The promoter regions in the primers are underlined. Because of low PCR yield, the fragment was cloned into pGEM-T-Easy vector (Promega, Madison, WI) and re-amplified. After PCR amplification, digoxigenin-labeled (Roche Diagnostics, Indianapolis, IN) antisense and sense transcripts were transcribed using T7 (New England Biolabs, Ipswich, MA) and T3 (Promega, Madison, WI) RNA polymerase, respectively. Following *in situ* hybridization, embryos were cleared in benzyl alcohol:benzyl benzoate (BABB) 2:1 and mounted in modified GMM mounting media (100 mL Canada Balsam, Sigma-Aldrich, St. Louis, MO; + 10 mL methyl salicylate, Sigma-Aldrich, St. Louis, MO) and photographed on a Nikon AZ100M microscope equipped with a Nikon DS-Fi1 camera.

### Data analysis

Raw data from the microarray analysis was mean-centered and quantile-normalized to normalize gene expression distributions across the different samples. The data was then Log_2_-transformed. Batch effects from the different biological replicates were removed using Partek Genomics Suite v 6.3 (http://www.partek.com/) and residual variance was analyzed by Principal Components Analysis (PCA) (Figure S1). Then the data was subjected to two-way ANOVA to study the effect of the developmental stages, treatment and their interactions. The development stages had the maximum effect on the gene expression, hence one-way ANOVA (*P*≤0.01) was used to identify genes altered by treatments at individual developmental stages. Venn diagrams were generated to illustrate the overlapping genes among the four different time points (*P*≤0.01, absolute fold change ≥|±1.4|). For the Hierarchical clustering, unsupervised hierarchical clustering was performed using Pearson correlation algorithm for the gene tree and Spearman for the developmental stages (*P*≤0.005).

### Biological function inference via pathway analysis and tissue enrichment

The corresponding human homologues to the differentially expressed zebrafish genes (*P*≤0.01, fold change ≥|±1.4|) were identified using ZFIN (http://zfin.org/) and Ensembl (http://www.ensembl.org). Gene ontology (GO) annotation biological processes enrichment of the estrogen responsive human homologues was performed by using Pathway Studio (Ariadne, MD). Fisher’s Exact test was used to calculate the *p*-value of each functional categories; *P*≤0.05 was considered significant. 

Zebrafish estrogen responsive gene expression locations were categorized according to ZFIN-Anatomy functional analysis at NIH-DAVID bioinformatics platform (http://david.abcc.ncifcrf.gov/tools.jsp). Fisher’s Exact test was used to calculate the *P*-value of the functional categories; *P* ≤0.05 was considered significant. 

### Comparison of estrogen-regulated gene expression between embryos and adult male fish

Data sets of estrogen-induced gene expression in male adult zebrafish were obtained from the GEO database (Gene Expression Omnibus; http://www.ncbi.nlm.nih.gov/geo/) (GEO accession # GSE27707). Series matrix.txt files with the log_2_-normalized ratios for all samples were downloaded and One-way ANOVA was used for the statistical analysis of the treatment groups and control groups. Benjamini-Hochberg false discovery rate correction (FDR) was applied to the raw *p*-value. FDR *q*-value ≤0.01 and fold change ≥|±2.0 | were chosen as a cut off for the differentially expressed genes. 

## Results

### Distinct gene expression profiles are regulated by E2 during early embryonic zebrafish development

**Figure 1:**
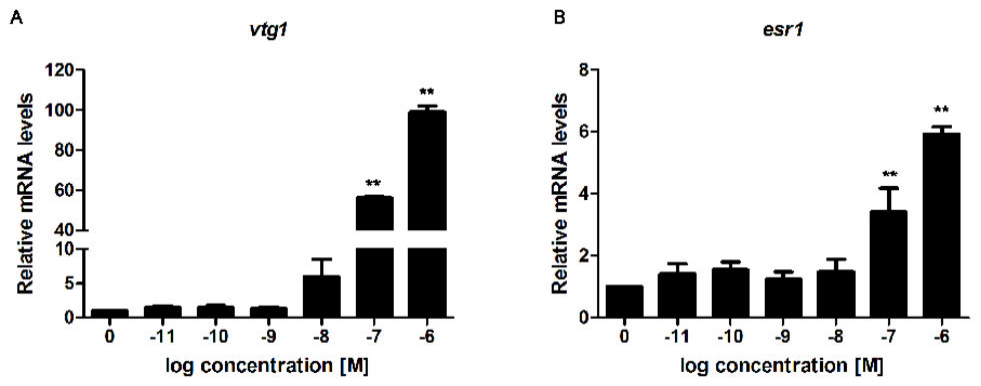
Dose-response curves of *vtg1* and *esr1* expression in zebrafish embryos. Zebrafish embryos were treated with increasing doses of E2 continuously for 4 days and the mRNA expression levels were determined by RT-qPCR. (A) Relative *vtg1* mRNA expression. (B) Relative *esr1* mRNA expression. Asterisk denotes significant differences (**P<0.01; unpaired Student’s t-test compared to the controls; n=2 biological replicates; 3 technical replicates within each biological replicate). Abbreviations *vtg*1: *vitellogenin 1* and *esr1*: *estrogen* receptor *1*.

To increase the understanding of how E2 acts on early zebrafish development, we performed transcriptomic analysis of whole embryos at different developmental time points. First to determine which dose of E2 to use for the microarray experiments, we performed dose response assessment of expression of the known estrogen targets *vtg1* and *esr1* by qPCR. Wild type zebrafish embryos were treated with E2 at concentrations ranging from 0.01 nM to 1 μM from 3 hpf to 4 dpf with daily media exchange. We have previously shown that 1 μM E2 is the highest concentration that zebrafish embryos can tolerate without showing obvious phenotypic abnormalities [19], thus we did not investigate higher concentrations. Embryos were pooled and collected at 4 dpf for RT-qPCR. Both *vtg1* and *esr1* expression were significantly induced by E2 treatment at 100 nM and maximally induced at 1 μM (Figure 1), thus we chose to perform the microarray at 1 μM to have an as high as possible E2-induced level of transcription. Furthermore, to determine the E2 uptake in the embryos, we exposed the embryos to 1 μM E2 from 3 hpf, collected the embryo media at 1, 2, 3 and 4 days of treatment and used UV-HPLC to assess the E2 levels remaining in the media (Figure S2). E2 uptake increased somewhat during the first 3 days of zebrafish development, and markedly increased at 4 dpf (21.6 ± 5.4% , 28.0 ± 12.0%, 31.7 ± 2.9% and 66.3 ± 3.2% E2 absorption at 1, 2, 3 and 4 dpf, respectively). 

**Figure 2:**
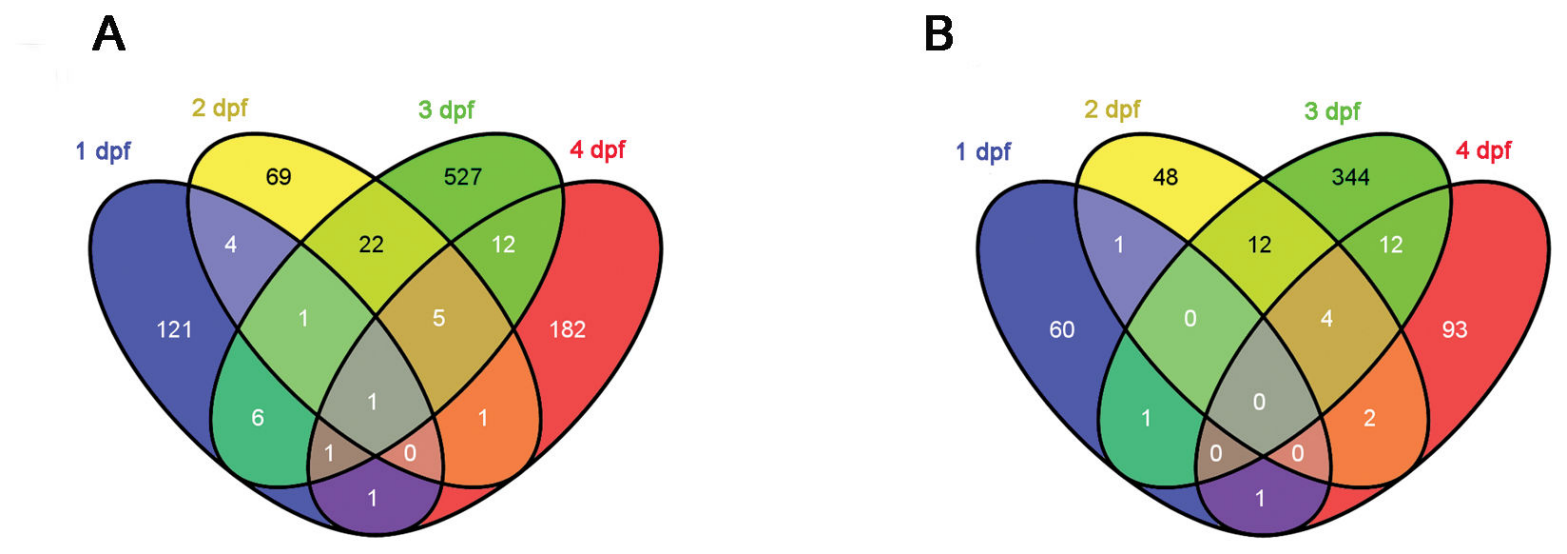
Distinct sets of genes are regulated by E2 during different times of zebrafish development. (A) Venn diagram illustrating the number of differentially expressed genes (*P*≤0.01, fold change ≥|±1.4|) that were regulated in common at the different time points. (B) Venn diagram of the human homologues of gene transcripts from (A).

We then performed the microarray analysis of whole-genome gene expression changes at the different developmental time points. A total of 28 arrays, probed with cDNA prepared from 3 biological replicates for each control and E2 treated fish at 1 and 2 dpf, and 4 biological replicates for 3 and 4 dpf was used for the transcriptome analysis. Transcriptome profiles identified 298, 219, 1016 and 444 probes significantly altered by E2 treatment at 1, 2, 3 and 4 dpf, respectively (P≤0.01, absolute fold change ≥|±1.4|) (Gene List in File S1). Out of these, 136 genes were successfully annotated at 1 dpf; 104 genes at 2 dpf; 576 genes at 3 dpf; 204 genes at 4 dpf (Table S2). The annotated genes were further sorted by fold change. The top 15 up and down regulated genes upon E2 treatment for each time point are shown in Tables S3-S6. When comparing individual gene expression changes at each time point, it was striking that distinct sets of genes were up or down-regulated by E2 at the different time points. Venn diagram analysis showed that only *vtg1* expression was co-regulated by E2 treatment at all four time points (P≤0.01, fold change ≥|±1.4|) (Figure 2A). The expression of 6 genes was co-regulated at 1 and 2 dpf, 9 genes at 1 and 3 dpf, 3 genes at 1 and 4 dpf, 29 genes at 2 and 3 dpf, 7 genes at 2 and 4 dpf, and 19 genes at 3 dpf and 4 dpf. Co-regulated genes among different time points are shown in Table S7. 

**Figure 3:**
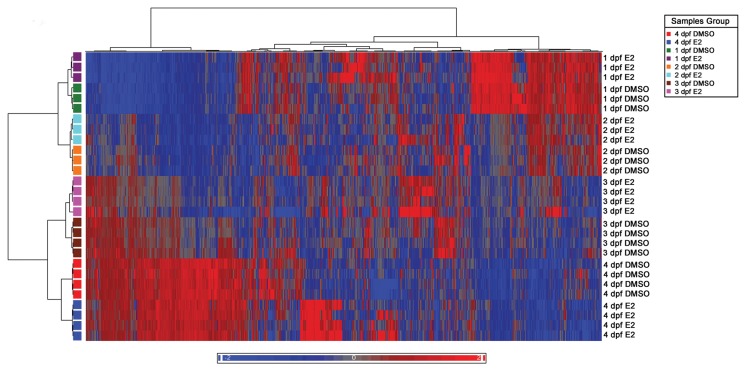
Clustering of gene expression profiles of E2 and vehicle treatment groups at different time points. The colored cells show the mean expression level of the biological replicates at each time point (1 dpf and 2 dpf: n=3; 3 dpf and 4 dpf: n=4). Red cells represent up-regulated genes, blue cells represent down-regulated genes and black cells represent unchanged expression levels between E2 and vehicle treated groups (*P*<0.005).

Then a Hierarchical clustering analysis of the significantly altered probes (*P*≤0.005) was performed using the Pearson correlation algorithm. For this clustering we used a *P*-value cutoff at *P*≤0.005 at which a clear developmental pattern of the differentially expressed genes was evident (Figure 3). Besides developmental changes, E2 treatment altered expression of a smaller set of genes at each developmental time point (Figure 3). Table S2 details the numbers of altered probes and genes at *P*-value cut-offs at 0.01 and 0.005. 

**Figure 4:**
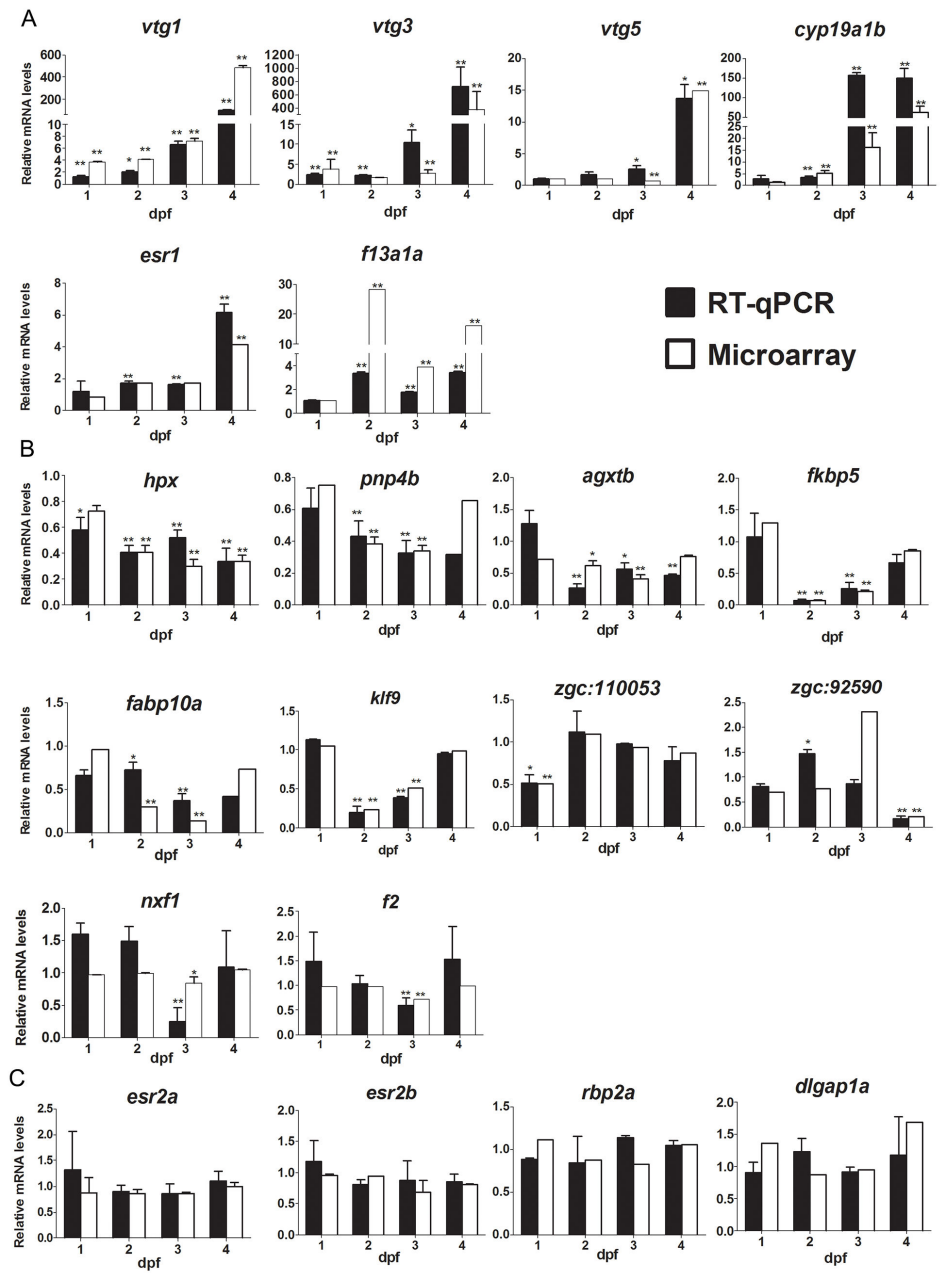
Comparison of E2 regulated genes analyzed by microarray or RT-qPCR. (A) Relative mRNA expression of the up-regulated genes *vtg1*, *vtg3*, *vtg5*, *esr1, cyp19a1b* and *f13a1a* at different time points as determined by RT-qPCR and microarray analysis. (B) Relative mRNA expression of the down-regulated genes *hpx*, *fkbp5*, *fabp10a*, *agxtb*, *pnp4b*, *nlf1*, *f2*, *klf9*, *zgc: 92590* and *zgc: 110053* at different time points as determined by RT-qPCR and microarray analysis. (C) Relative mRNA expression of the non-changed genes *esr2a*, *esr2b*, *rbp2a* and *dlgap1a* at different time points as determined by RT-qPCR and microarray analysis. White bars represent microarray results and black bars RT-qPCR results. Asterisk denotes significant difference (*P<0.05, **P<0.01; unpaired Student’s t-test compared to the controls), n≥3 biological replicates except for genes *fabp10a*, *agxtb* and *zgc:110053* which were 2 biological replicates ; each replicate consists of 30 pooled embryos. Abbreviations *vtg*1: *vitellogenin 1*; *vtg*3: *vitellogenin 3*; *vtg*5: *vitellogenin 5*; *esr1*: *estrogen* receptor *1*; *esr2a*: *estrogen* receptor *2a*; *esr2b*: *estrogen* receptor *2b*; *cyp19a1b*: *cytochrome* *P450*, family *19, subfamily* *A*, *polypeptide 1b*;　*f13a1a: coagulation* *factor* *XIII, A1* *polypeptide* *a*, *tandem* *duplicate 1; hpx*: *hemopexin*; *fkbp5*: *FK506* *binding* protein *5*; *fabp10a*: *fatty* *acid* *binding* protein *10a*; *agxtb*: *alanine-glyoxylate* *aminotransferase* *b*; *pnp4b*: *purine* *nucleoside* *phosphorylase 4b*; *nxf1*: *nuclear* *RNA* *export* factor *1*; *klf9*: *krueppel-like* *factor 9*; *f2: coagulation* *factor* *II* (thrombin)*; rbp2a*: *retinol* *binding* protein *2a* and *dlgap1a*: *discs, large* (Drosophila) *homolog-associated* protein *1a*.

Selected genes were validated with RT-qPCR (Figures 4 and S3). Overall, there was a high concordance between the microarray and the RT-qPCR results. For the up-regulated genes, qPCR confirmed the regulation of *vtg1, vtg3, vtg5, cyp19a1b, esr1, and f13a1a* by E2, although at one time point (2 dpf) we only detected the up-regulation of *vtg3* by RT-qPCR and not by microarray (Figure 4A). Expression of *esr1* was significantly up-regulated in RT-qPCR at 2, 3 and 4 dpf, but only at 4 dpf in microarray datasets (Figure 4A). The fold changes between RT-qPCR and microarray varied slightly when the induction factors were very high, but in general the results showed high concordance. For the down-regulated genes, the gene expression changes measured by RT-qPCR were also consistent with results obtained by microarray analysis. Expression of *hpx, agxtb, fabp10a, fkbp5*, *klf9*, *pnp4b, zgc:110053, nxf1, f2*, and *zgc:92590* were significantly down-regulated based on both microarray and RT-qPCR (Figure 4B). The expression of the *esr2a*, *esr2b*, *dlgap1a* and *rbp2a* genes were also confirmed by RT-qPCR, and the expression levels were not significantly changed at any of the four time points, which was in concordance with the microarray data (Figure 4C). The 18S rRNA gene was used as a reference gene for all RT-qPCRs. The expression of this gene was not influenced by E2 at these developmental stages (Figure S4). In summary, the validated RT-qPCR results showed high concordance with microarray results (Figure S3). 

### Similar biological processes are regulated by E2 at different developmental stages

In order to profile E2-regulated biological functional processes, Gene Ontology (GO) enrichment analysis was performed. Due to the lack of zebrafish pathway analysis databases, the human homologues to the zebrafish estrogen-responsive genes were identified through the ZFIN (http://zfin.org/) and Ensembl zebrafish Zv9 databases (http://www.ensembl.org/Danio_rerio/), followed by GO enrichment using Pathway Studio mammalian database. Approximately half of the annotated differentially regulated genes were successfully identified with human homologues (64 (47.06%) at 1 dpf, 68 (65.38%) at 2 dpf, 374 (64.93%) at 3 dpf and 112 (54.90%) at 4 dpf). The human homologues to the 15 most up- and down-regulated estrogen-responsive genes at different time points are shown in Tables S3-S6. A Venn diagram in Figure 2B shows human homologues of differentially expressed genes regulated in common at the different time points and Table S7 details the overlapping human homologues. 

GO annotation analysis based on the human homologues revealed a significant enrichment in several biological processes after E2 exposure at the four time points (Table 1). The broad functional categories included metabolic process, transcription, transport, and signal transduction. Also, genes for the phosphorylation, immune response and multicellular organismal development categories were co-regulated at all four time points. Apoptosis and cell proliferation categories were enriched from the differentially expressed genes at 3 and 4 dpf (Table 1). Under the broad functional categories, more specific sub-categories were identified (Tables S8-S12). Several of the sub-categories were in common across the four developmental stages. Hormone biosynthetic process, steroid signaling pathway and response to estrogen stimulus validated the estrogenic effects during zebrafish development after E2 treatment. To summarize, although distinct sets of genes were regulated by E2 at different time points, the biological processes that these genes affected were similar. 

**Table 1:** Gene ontology biological process functional groups enrichment based on human homologues of zebrafish E2-regulated genes.

Category*	**1 dpf**		**2 dpf**		**3 dpf**		**4 dpf**	
	Percent (%)	p-value	Percent (%)	p-value	Percent (%)	p-value	Percent (%)	p-value
Metabolic process	15.63	**1.43E-02**	28.13	**3.02E-02**	24.93	**1.14E-17**	38.32	**2.53E-06**
Regulation of transcription	10.94	**1.24E-02**	12.50	**3.12E-02**	10.03	**3.93E-03**	12.15	**2.13E-02**
Transport	17.19	**5.01E-04**	23.44	**2.04E-02**	18.70	**2.45E-13**	28.04	**9.82E-05**
Signal transduction	28.13	**6.69E-05**	17.19	**4.02E-02**	15.72	**3.62E-04**	18.69	**6.83E-03**
Response to chemical stimulus	--	--	7.81	**9.46E-05**	3.52	**1.12E-06**	6.54	**9.02E-06**
Apoptosis	3.13	4.33E-01	6.25	6.52E-02	4.34	**7.48E-03**	5.61	**3.51E-02**
Cell proliferation	--	--	3.13	2.04E-01	2.98	**5.37E-03**	4.67	**1.12E-02**
Phosphorylation	7.81	**1.56E-05**	3.13	**2.03E-04**	5.42	**1.07E-04**	3.74	**2.03E-02**
Multicellular organismal development	7.81	**1.09E-02**	4.69	**1.93E-02**	4.61	**2.67E-02**	3.74	**4.86E-03**
Immune response	4.69	**2.00E-03**	4.69	**1.82E-02**	2.17	**1.76E-02**	3.74	**1.73E-02**

* Category represents main functional group, but p-value may represent subgroups of the main groups.

Bold p-values represent statistically significant categories (p<0.05).

### E2 targets various tissues during embryonic zebrafish development

To infer the spatial expression patterns of the E2 responsive genes in the embryos, we performed knowledge-based tissue enrichment analysis using ZFIN_Anatomy functional analysis tool at NIH DAVID bioinformatics microarray analysis platform (http://david.abcc.ncifcrf.gov/tools.jsp). E2-responsive genes lists were uploaded to NIH DAVID website, followed by tissue enrichment analysis based on previously published tissue-specific gene expression information in ZFIN public database. Enriched tissue categories from the E2-responsive genes represent putative estrogen-responsive tissues. The numbers of genes identified by NIH DAVID and enriched using ZFIN_Anatomy tissue-specific analysis tool are listed in Table S13. E2-responsive genes were significantly enriched in the brain at all four time points although at 3 dpf, ventral telencephalon, which is a substructure in the brain, was enriched (Table 2). Liver, pancreas, and reproductive organ categories emerged at 2, 3 and 4 dpf. In addition, the retinal photoreceptor layer category was enriched at 1 dpf, and the intestinal bulb category was enriched at 2 and 3 dpf. E2-responsive genes were enriched in the kidney and pronephric duct categories at 3 dpf, and in the neuromast category at 4 dpf. To confirm the tissue specific expression, we performed *in situ* hybridization using an anti-sense RNA probe of *vtg4*, which was the most highly E2-activated gene in the whole microarray, activated 1,218 times at 4 dpf (Table S6). *Vtg4* RNA was predicted to be expressed in the liver by ZFIN_Anatomy functional analysis tool. As shown in Figure S5, E2 strongly induced *vtg4* expression in the liver of DZ embryos at 4 dpf, compared to DMSO-treated embryos. No signal was detected in the embryos hybridized with sense *vtg4* RNA probe in either E2- or DMSO-treated groups.

**Table 2:** ZFIN anatomy functional chart of E2 responsive genes enriched by NIH DAVID analysis tool.

**Term**	**Count**	**%**	**p-value**	**Genes**
**1 dpf**				
Brain	9	9.18	3.20E-02	*gria2a, gria3a, oprd1a, grk7a, ppp1r1c, ptprn2, angptl1, lrrc4c, scn4ba, gria4a, epd*
Retinal photoreceptor layer	3	3.06	1.20E-02	*grk7a, zgc:112320, ntm*
Cephalic musculature	3	3.06	5.80E-03	*smyhc2, myhb, hspb8*
**2 dpf**				
Brain	9	13.2	1.50E-02	*bsk146, rgs2, nr1d2a, hmgcs1, vipr1, tcf7l2, rxraa, epd, cyp19a1b*
Liver	8	11.8	1.70E-02	*pnp4b, rgs2, hpx, pglyrp2, hmgcs1, vtg1, cyp19a1b, fabp10a*
Pancreas primordium	3	4.41	2.30E-03	*hsd11b2, spon1b, atp1a3a*
Intestinal bulb	4	5.88	8.80E-03	*bcmo1, pnp4b, rgs2, zgc:110176*
YSL	6	8.82	1.40E-02	*nr0b2a, mxtx1, hmgcs1, gnsb, arl5c, nfkbiaa*
Ovary	4	5.88	1.90E-02	*amh, vtg1, vipr1, cyp19a1b*
Testis	5	7.35	9.70E-04	*amh, vtg1, vipr1, fabp10a, cyp19a1b*
**3 dpf**				
Ventral telencephalon	5	1.26	1.40E-03	*fgf19, cadm4, nr4a1, etv1, cyp19a1b*
Liver	40	10.1	3.00E-08	*cyp1b1, mre11a, lhcgr, hmgcs1, kmo, si:ch211-93f2.1, il17rd, scn1ba, atp2b1b, cyp19a1b , fabp10a, ttr, sult2st1, myd88, zgc:103559, vtg3, vtg1, vtg5, unc45a, shbg, cebpd, hkdc1, fa2h, atp7a, sall4, pnp4b, rgs2, uox, hpx, f2, ghrl, ripk2, srd5a2a, zgc:92111, zgc:153921, eaf2, nr5a5, acad11, lipc, fabp6, c3b*
Pancreas	10	2.53	6.80E-04	*cpa4, ins, ctrb1, neu3.3, ghrl, zgc:92111, gcga, zgc:66382, try, ela3l*
Intestinal bulb	10	2.53	5.20E-03	*sult2st1, dab2, pnp4b, myd88, rgs2, neu3.3, si:ch211-93f2.1, srd5a2a, nr5a5, acad11*
Kidney	10	2.53	9.30E-03	*cyp1b1, slc2a11l, bcl2, slc13a1, slc26a6l, ripk2, eaf2, atp2b1b, illr4, fabp6*
Pronephric duct	16	4.04	5.70E-02	*hkdc1, ms4a17a.5, fa2h, zgc:101040, sypl2a, atp1a3b, kmo, slc20a1a, cep70, tnfrsf1a, dab2, sall4, myd88, slc13a1, ahcyl2, ip6k2*
Ovary	12	3.03	3.30E-03	*cyp1b1, zp3, lhcgr, atp2b1b, cyp19a1b, amh, bcl2, vtg3, ghrl, vtg1, nr5a5, vtg5, fabp6*
Testis	9	2.27	1.10E-02	*amh, cyp1b1, lhcgr, ghrl, vtg1, nr5a5, atp2b1b, vtg5, cyp19a1b, fabp10a*
**4 dpf**				
Brain	12	8.45	3.20E-02	*pgr, rarab, ahr1a, irak3, bdnf, rgs2, rnaset2, eif4a2, lhbeta1, cdk5, disc1, cyp19a1b*
Liver	19	13.4	1.70E-06	*slc43a1a, zgc:174260, lhbeta1, esr1, zgc:113054, ahsg, cpn1, cyp19a1b ,bdnf, serpina7, rgs2, atic, hpx, rnaset2, vtg3, vtg4, vtg1, vtg2, rpia, lipc, vtg5*
Pancreas	6	4.23	6.00E-04	*zgc:92590, gip, ins, rwdd3, gcga, amy2a*
Neuromast	4	2.82	9.80E-03	*bdnf, vtg3, zgc:56382, sb:cb252*
Ovary	8	5.63	3.10E-04	*amh, rnaset2, lhbeta1, vtg3, esr1, vtg1, vtg2, vtg5, cyp19a1b*
Testis	6	4.23	1.90E-03	*pgr, amh, rnaset2, lhbeta1, vtg1, vtg5, cyp19a1b*

**Figure 5:**
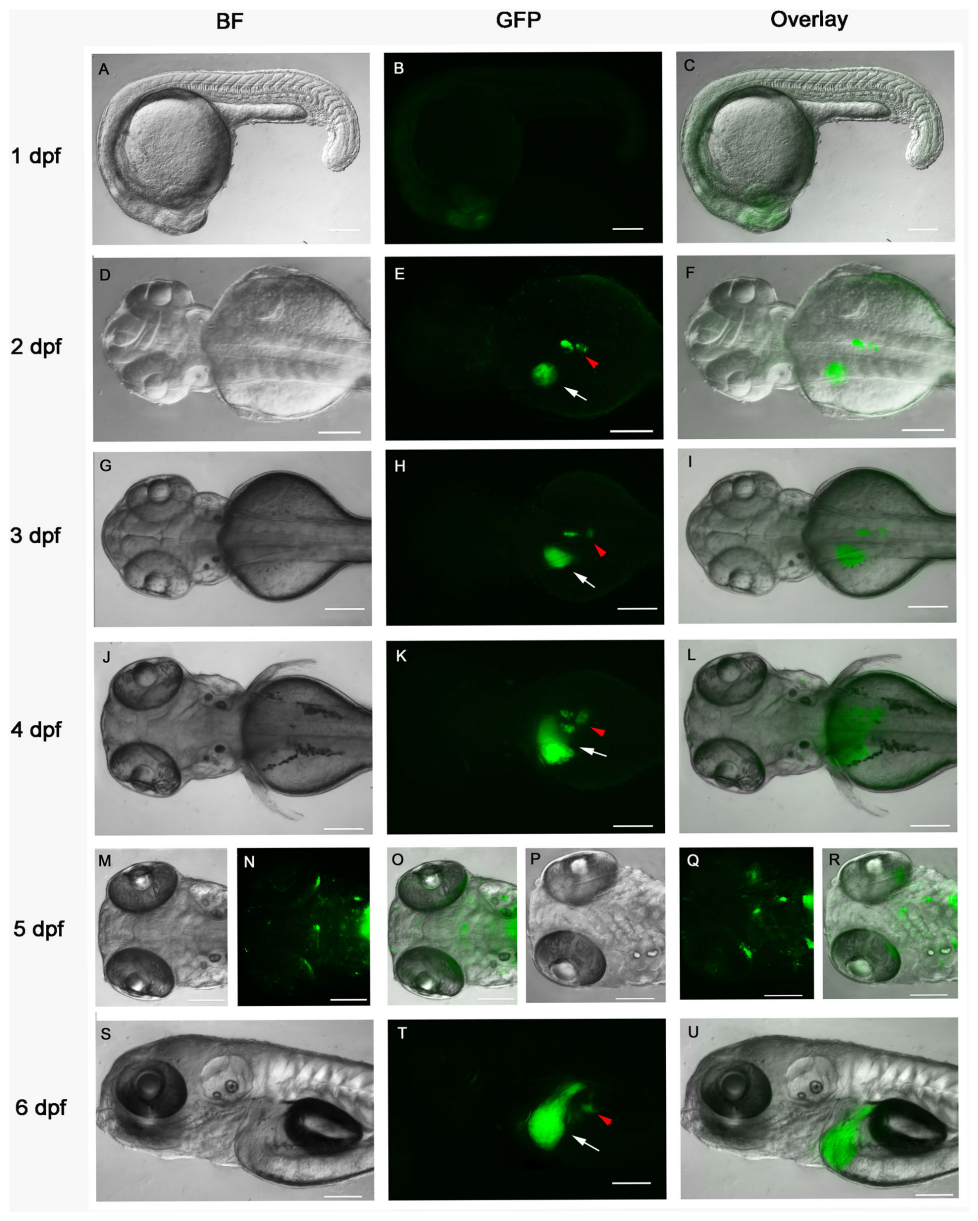
Developmental dynamics of E2 responsive tissues in *Tg*(5xERE:GFP) transgenic fish. Zebrafish larvae were treated with 1 μM E2 (in 0.1% DMSO) from 3 hpf and imaged at 1 dpf (A-C), 2 dpf (D-F), 3 dpf (G-I), 4 dpf (J-L), 5 dpf (M-R) and 6 dpf (S-U). *Arrows* (white) indicate the liver; *arrowheads* (red) indicate the pancreas. A, D, G, J, M, P and S, bright-field images; B, E, H, K, N, Q and T corresponding GFP fluorescence images; C, F, I, L, O, R and U, overlay of bright-field and GFP images. A-C and S-U, lateral view; D-O, dorsal view; P-R, ventral view; anterior to the left. *Scale* *bars*, 100 μm.

We then used a transgenic reporter zebrafish line *Tg*(*5xERE:GFP*), expressing GFP driven by 5×*ERE*, to visualize the developmental dynamics of E2-responsive tissues [17]. Live E2-treated fish embryos were observed from 3 hpf to 6 dpf for GFP expression. In the absence of E2 treatment, fluorescence signal was detected before 1 dpf. To determine whether this signal was caused by maternal load of GFP or by zygotic transcription/translation, we crossed *Tg*(*5xERE:GFP*) fish with wild type DZ fish. The embryos from female *Tg*(*5xERE:GFP*) fish crossed with male wild type DZ fish expressed a similar fluorescence signal to the homozygous *Tg*(*5xERE:GFP*) embryos (Figure S6 A-D). Conversely, when crossing male *Tg*(*5xERE:GFP*) fish with female DZ fish, no fluorescence was detected in the embryos (Figure S6 E-H). Thus, these results suggested that the initial fluorescence of the *Tg*(*5xERE:GFP*) embryos represented maternal load of GFP expression activated by endogenous estrogens in the female fish. At 1 dpf, the maternal GFP fluorescence had faded and the zygotic GFP expression appeared mainly in the head region after E2 treatment but not in untreated embryos (Figure 5 A-C, and results not shown). In the presence of E2, strong GFP fluorescence was detected from 2 dpf in the presumptive liver progenitor cells as well as pancreas, and persisted as the liver and pancreas developed (Figure 5 D-U). GFP-positive cells were also detected in the brain and heart valves at 4 dpf, but at very low levels. At 5 and 6 dpf, the GFP expression was more visible in the brain, pre-optic nerves, hair cells, heart valves, liver and pancreas (Figure 5 J-U). The GFP expression at 5 dpf was similar to the previous report on these transgenic fish [17], except for the pancreas expression not previously described. To confirm that the endocrine pancreas is one of the E2 target tissues, we crossed *Tg*(*5xERE:GFP*) with *Tg*(*ins:mCherry*) transgenic fish, the latter expressing mCherry driven by insulin promoter. The embryos were treated with E2 and fluorescence was observed daily. From 2 dpf to 6dpf, a co-localization of mCherry and GFP fluorescence was observed in the pancreatic islets (Figure S7 and results not shown). 

To conclude, according to the target tissue enrichment analysis, endogenous E2-responsive genes were predicted to be expressed in the liver, pancreas and at various locations of the brain during early zebrafish development (Table 2). This correlates to the regions of GFP expression in *Tg*(*5×ERE:GFP*) transgenic fish after E2 induction (Figure 5). The results are also in accordance to the biological pathway analysis showing that the most regulated process is the one of metabolism, presumably taking place to a large extent in the liver. We thus propose from these findings that liver, pancreas and brain are sensitive organs for estrogen treatment, or exposure to other estrogenic compounds, during early zebrafish development.

### Comparison of estrogen signaling between embryonic and adult zebrafish

**Figure 6:**
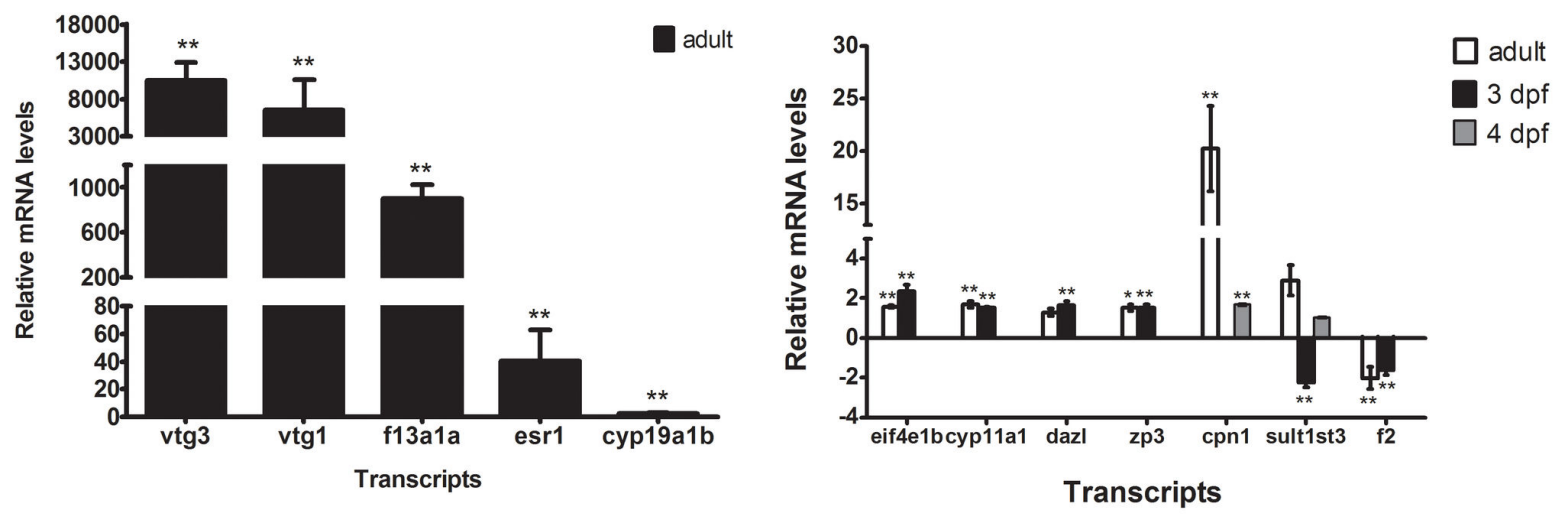
Validation of co-regulated differentially expressed genes in embryos and adult males using RT-qPCR. (A) Relative mRNA expression of the up-regulated genes *vtg1*, *vtg3, esr1* , *cyp19a1b* and *f13a1a* in adult males upon E2 treatment. (B) Relative mRNA expression of the *genes* *eif4e1b, cyp11a1, dazl, zp3, cpn1, sult1st3* and *f2* in 3 dpf and 4 dpf larvae as well as in adult males upon E2 treatment. **P<0.01; unpaired Student’s t-test compared to the controls. Abbreviations *eif4e1b: eukaryotic* *translation* *initiation* factor *4e 1b, cyp11a1* (cytochrome P450, subfamily XIA, polypeptide 1)*, dazl: deleted* *in* *azoospermia-like, zp3: zona* *pellucida* *glycoprotein 3, cpn1*(carboxypeptidase N, polypeptide 1)*, sult1st3: sulfotransferase* family *1, cytosolic* *sulfotransferase 3*.

Another study has reported whole organism estrogen responsive genes identified in E2-treated male adult zebrafish by microarray analysis [13]. To investigate whether there were any E2 target genes co-regulated in adult zebrafish and embryos in response to E2, we compared our gene lists to those of Lam and colleagues (GEO accession # GSE27707). A subset of genes were co-regulated among our significantly up-regulated and down-regulated genes (*P*≤0.01, fold change ≥|±1.4|) and estrogen responsive genes in male adult fish (*q*≤0.01, fold change ≥|±2.0|) (using the same fold induction as in the Lam et al. publication [13]) (Table 3). The expression of *vtg1* was co-activated in male adult fish and all four embryonic stages whereas *vtg3* was up-regulated in all the stages except at 2 dpf. Expression of *cyp11a1*, *eif4e1b*, *dazl* and *zp3* were up-regulated in 3 dpf embryos and adult males, while expression of *esr1, atic* and *cpn1* were up-regulated in 4 dpf embryos and adult males (Table 3). The down-regulated genes in common in both embryos and adult males were *sult1st3, f2* and *zgc:56382* (Table 3). The expression of selected co-regulated genes was validated in embryos and adult fish by RT-qPCR (Figure 4, 6A and B). The known estrogenic markers *vtg1*, *vtg3* and *esr1*, as well as new genes *cpn1, eif4e1b*, *cyp11a1*, and *zp3* were all significantly up-regulated by E2 in the adult fish and embryos (Figure 4 and 6). Expression of *dazl* was significantly up-regulated in the 3 dpf embryos but not in the adult males. Expression of *sult1st3* was significantly down-regulated in the 3 dpf embryos but not in 4 dpf embryos and in adult males, which was different from what the microarray analysis predicted. Expression of *f2* was down-regulated in both 3 dpf embryos and adult males (Figure 6B). In addition to these co-regulated genes, we also investigated the expression of *f13a1a* and *cyp19a1b* using RT-qPCR, since their expression was highly up-regulated in the E2-treated embryos (Figure 4), and showed that both *f13a1a* and *cyp19a1b* levels were also elevated (Figure 6A). To summarize, although E2 to a large extent regulates the expression of distinct sets of genes in embryonic and adult fish, a limited number of co-regulated genes were identified. 

**Table 3:** Co-regulated estrogen-responsive genes in embryos and male adult fish from [13].

Up-regulated genes				
Embryos	1dpf	2dpf	3 dpf	4 dpf
Adult	*vtg1, vtg3*	*vtg1*	*vtg1, vtg3, cyp11a1, eif4e1b, daz1, zp3*	*vtg1, vtg3, esr1, cpn1, atic*
Down-regulated genes				
Embryos	1dpf	2dpf	3 dpf	4 dpf
Adult			*sult1st3, f2, opn1mw3*	*sult1st3, zgc:56382*

## Discussion

### E2 regulates estrogen-responsive genes in a stage-dependent manner

We identified E2-responsive genes that were regulated at 1-4 dpf during zebrafish development through transcriptomic analysis. The microarray data predicted that E2 activated transcription of a distinct set of genes in a stage and tissue specific manner during zebrafish development. 

From hierarchical clustering and Venn diagram analysis, we found that most of the differentially expressed genes regulated by E2 treatment were distinct at different stages during zebrafish development (Figures 2 and 3). At 1 dpf, among the top up- and down-regulated genes were genes that encode proteins involved in ubiquitination (*dcaf13*), phosphorylation (*ptprc*, *ppp1r1c* and *dusp3*), tissues development (*hoxd12a*, *znf644* and *opn1lw2*), immune system (*igsf21b*, *nitr3d*), and synapse function (*ddc*, *gria4a* and *grid2*) (Table S3). At 2 dpf, the most highly activated (28 fold) gene transcript was *f13a1a*, which encodes coagulation factor VIII, and the most down-regulated (15 fold) gene transcript was *fkbp5*, an immunophilin involved in protein folding and trafficking (Table S4). At 3 dpf, the *cyp19a1b* gene showed the most highly activated transcription following E2 treatment (35 fold) (Table S5). Other activated transcripts were transcription factors (*batf*, *hoxb9a*, *ccdc37* and *morc3b*) and ubiquitin-related genes (*ubn2* and *exosc6*). The top down-regulated transcripts at 3 dpf were two fatty acid binding proteins (*fabp10* and *fabp6*), and other down-regulated genes encoded proteins involved in transport, including the heme transporter *hpx*, the ammonium transporters *rhcga* and *rhcgb*, and *slc6a*, which transport amino acids in the kidney. Finally, the most up-regulated transcripts at 4 dpf were clearly the *vtgs 4, 3, 1, 2* and *5* (1218, 646, 522, 44 and 15 fold up-regulated by E2, respectively) (Table S6). The most highly down-regulated gene transcripts at this stage were *pfkfb4* (9 fold), a hypoxic induced kinase/phosphatase, and *upp2* (6 fold), which is a uridine phosphatase. The fold changes of E2-altered genes were much higher at 4 dpf than the other three time points, which might be caused by the higher E2 levels in the embryos (Figure S2). 

Although E2 regulated distinct sets of genes at different time points during zebrafish development, some differentially expressed genes were in common at several time points. The well-known estrogenic biomarkers *vtg1*, *vtg3, vtg5, esr1*, and *cyp19a1b* were found to be E2-target genes in our study, confirming that our results are in line with previously published results [20-23]. Expression of *vtg1* and *vtg3* was not only up-regulated by E2 at embryonic stages but also in male adult fish, as published previously (Figure 4, 6A and [13]), verifying the status of these genes as reliable estrogenic biomarkers. 

In addition to the well-known estrogenic biomarkers, we identified other E2-regulated transcripts at various zebrafish developmental stages to be potential biomarkers for estrogen signaling. For example, expression of *coagulation factor XIII, A1 polypeptide a* (*f13a1a*) was highly up-regulated by E2 treatment in 2, 3, 4 dpf embryos as well as male adults (Table S4-S6, Figure 4 and 6). Furthermore, transcription of *ependymin* (*epd*), encoding a brain extracellular glycoprotein involved in memory and neuronal regeneration (further discussed below), was down-regulated by E2 at three time points. Transcription of *anti-Mullerian hormone (amh*), encoding a steroidogenic enzyme, was induced at three time points. In previous studies, *amh* was reported to be induced in both prepubertal and adult rats exposed to methoxychlor, a previously commonly used pesticide, now banned in the US because of its estrogenic disrupting properties [24,25]. The transcripts of *regulator of G-protein signaling 2* (*rgs2*) and *hemopexin* (*hpx*) were also highly altered by E2 treatment, as described above. HPX protein levels are reportedly reduced in estrogen-treated menopausal women [26], which is consistent with the decrease of *hpx* transcripts after E2 treatment in our study. Genes that were regulated at two embryonic time points include *ankyrin repeat* domain *9* (*ankrd9*)*, FK506 binding* protein *5* (*fkbp5*), *fatty acid binding* protein *10a* (*fabp10a*), *purine nucleoside phosphorylase 4b* (*pnp4b*), *proopiomelanocortin a* (*pomca*) and *dopa decarboxylase* (*ddc*) (Table S3-S6). Further investigations will be required to understand the role of estrogen regulation of these genes during development. 

Comparison of the E2 target genes between embryonic and adult fish showed that the expression of a few genes were co-regulated, which indicates that the E2-regulated genes identified in our microarray analysis might be development specific. However, some co-regulated genes, such as *f13a1a* and *cyp19a1b*, were missed in the microarray comparisons, but identified by RT-qPCR, which could be explained by that the two microarray platforms were designed from different companies and contained different number of probes (16K vs. 44K) [13]. Nevertheless, the co-regulated genes of this study, including the known estrogenic markers *vtg1*, *vtg3*, *and esr1*, as well as the new markers *f13a1a* and *cpn1*, could potentially serve as biomarkers of estrogenic exposure to both embryos and adult fish*.*

### Estrogen signaling during early zebrafish development is tissue specific

Putative estrogen target tissues, in which E2-responsive genes have been reported to be expressed, were predicted by using ZFIN anatomy functional analysis at NIH DAVID bioinformatics platform (Table 2). Differentially expressed genes were enriched in brain, liver, and pancreas at 2-4 dpf, which is in accordance with the GFP-expressing tissues in the *Tg*(*5xERE:GFP*) transgenic fish (Figure 5 and [17]). Similar estrogen responsive tissues have also been reported by another group using a similar 3×ERE-Gal4ff/UAS-GFP double transgenic fish [27]. However, the latter fish model showed additional GFP expression in the muscle fibers in the somites at 4 dpf, a finding that was not made with the *Tg*(*5xERE:GFP*) fish [17,27]. Supporting a role for E2 in muscle, one of the GO categories enriched at 2-4 dpf was “muscle development” (Table S12).

In our tissue enrichment analysis, the tissue with the most E2-responsive genes, such as *fabp10a, pnp4b, hpx, rgs2*, and the *vtgs*, was the liver (Table 2). Liver is the major organ for metabolism, detoxification and homeostasis. In the *Tg*(*5xERE:GFP*) transgenic fish, liver expression of GFP can be detected after 35 hpf, which is in agreement with *in situ* hybridization of *ceruloplasmin* of developing liver [28], and liver-specific dsRed expression of transgenic zebrafish *Tg*(*lfabf:dsRed; elaA:EGFP*) [29]. Consistent with our results, the fish liver is a main target for both endogenous and exogenous estrogens, and the classical estrogen biomarkers in fish include the liver-specific genes *vtgs* and *esr1*. We found that the most highly up-regulated gene at 4 dpf was *vtg4*, which was predicted to be expressed in the liver at the same time point (Table 2). We also confirmed *vtg4* up-regulation in the liver by *in situ* hybridization (Figure S5). As a member of the *vtg* family, *vtg4* has been investigated in the adult liver [23,30]; however, no such study has been performed for developing embryos. 

Another tissue in which E2-responsive genes were predicted to be expressed was the pancreas. Clusters of gene transcripts were enriched in the pancreas at 2 dpf, 3 dpf and 4 dpf (Table 2). Similar to the mammalian pancreas, the zebrafish pancreas includes both an exocrine/duct compartment and endocrine part comprising alpha, beta, delta, epsilon and pancreatic polypeptide (PP) producing cells [31]. The endocrine pancreas is one of the major organs of zebrafish endocrine system secreting insulin, glucagon, PP, ghrelin and somatostatin [31]. Our enrichment predicted that transcription of *insulin (ins*), *ghrelin/obestatin preprohormone* (*ghrl1*) and *glucagon a* (*gcga*) were E2-regulated in the pancreas (Table 2). The endocrine pancreas was also an E2-responsive tissue in the *Tg*(*5xERE:GFP*) transgenic fish (Figure 5), which was confirmed by fluorescence co-localization in pancreatic islets of *Tg*(*ins:mCherry*)*/Tg*(*5xERE:GFP*) embryos after E2 treatment (Figure S7). These results suggest that estrogen receptors are present in the pancreas and that estrogen signaling plays a role in zebrafish pancreas development. Although no report has been published linking estrogens to pancreas function in zebrafish, the evidence for this connection in mammals is extensive. In particular the role of estrogens in regulation of proliferation, differentiation, and survival of β cells and of insulin synthesis and release has been described (reviewed in 32). 

Estrogen receptors, in particular ERβ (ESR2), are important for normal brain and behavior development in rodents. Whereas ERα (ESR1) is the predominant ER in the hypothalamus, controlling reproductive cycles, ERβ is expressed in the cerebral cortex, the hippocampus, the cerebellum and the dorsal raphe (reviewed in 3). In zebrafish, estrogenic regulation of GFP expression occurs at several locations, including the preoptic area, olfactory bulb and hypothalamus in the brain of the transgenic *Tg*(*5xERE:GFP*) fish ([17] and Figure 5). In accordance with special expression of ERE-induced GFP, the brain was one of the tissues that were predicted to have many enriched gene transcripts following E2 treatment. At the earliest time point studied, several subunits of the α*-amino-3-hydroxy-5-methyl-4-isoxazoleproprionate* (*AMPA*) *type glutamate receptors* were identified as E2 target genes. These receptors mediate the majority of fast synaptic glutamate transmissions, which are known to promote neuronal growth, retraction and elongation of glial processes, proliferation and differentiation of retinal progenitors and proliferation of cortical progenitors. Specifically, *gria2a*, *gria3a*, *gria4a* glutamate receptor subunits were regulated by E2. The AMPA receptors have previously been shown to be regulated by estrogen in rats and mice [33,34]. The *epd* transcript, encoding a protein involved in neuronal plasticity, neurobehavior (memory and aggression) and cold adaptation, and the neurobehavioral *disc1* transcript were also regulated by E2 treatment in embryonic zebrafish brain. The *disc1* gene has been associated with risk of schizophrenia, bipolar affective disorder and major depression [35]. Also *cyp19a1b* (aromatase B) was found to be regulated by estrogen in the brain at 2-4 dpf. Although in the tissue clustering, this gene was designated to several tissues, including liver [36], *cyp19a1b* has been reported to be brain-specific in zebrafish while *cyp19a1a* encodes aromatase in the ovary [37]. We did not detect any *cyp19a1b* mRNA in adult fish liver extracts by RT-qPCR (results not shown). More specifically, expression of *cyp19a1b* has been shown to be up-regulated by E2 in zebrafish radial glial cells [38]. 

A group of estrogen responsive genes were predicted to be expressed in kidney or pronephric duct, including the cytochrome P450 *cyp1b1*, the ATPases *atp2b1b* and *atp1a3b* (Ca^++^ and Na^+^/K^+^ transporting, respectively), fatty acid binding protein 6 (*fabp6*), and the solute carrier family members *slc2a11l, slc13a1, slc26a6l* and *slc20a1a. Cyp1b1* has been detected in the developing kidney during early murine development [39]. It metabolizes estradiol and plays an important role in normal embryonic development [40]. Renal dysfunction and inflammation associated with angiotensin II-induced hypertension of the mouse model are *cyp1b1* dependent [41]. In line with E2 regulation in the kidney and/or pronephric duct of the four transcripts that belong to genes of the solute carrier family, *slc2a11l* (glucose transporter), *slc13a1* (sodium/sulphate transporter), *slc26a6l* (anion transporter) and *slc20a1a* (phosphate transporter), one of the biological functional groups that were regulated by E2 was “Transport”, as discussed below.

Besides liver, brain, pancreas and kidney, the predicted tissue enrichment in Table 2 also includes cephalic musculature and retinal photoreceptor at 1 dpf, and intestinal bulb, testis and ovary at 2-4 dpf; however, in the 5×ERE-transgenic fish we failed to detect GFP expression in any of these organs at the early developmental stages (data not shown). Intestine, testis and ovary expression of GFP were, however, detected in the adult *Tg*(*5xERE:GFP*) transgenic fish (data not shown). Normal morphogenesis of ovary and testis does not initiate until 10 dpf [42,43], but some genes controlling sex differentiation like *amh* may be altered by E2 treatment or play other roles at earlier stages of zebrafish development. In addition, *Tg*(*5xERE:GFP*) fish showed weak GFP expression in the heart valves from 4 dpf, which is in agreement with observations from the *Tg*(*3×ERE-Gal4ff/UAS-GFP*) fish [17,27]. However, we did not obtain any enrichment of estrogen-responsive gene transcripts in the heart (Table 2). Finally, the tissue analysis predicts that estrogen regulates genes in neuromasts, cells that has been shown to depend on *esr2a* for their development [9]. Expression of brain-derived neurotrophic factor (bdnf), which is involved in development and maintenance of neuromasts [44], was up-regulated by E2. Although this gene has not been shown to be regulated by estrogen in zebrafish, many reports have described an E2 induction of *bdnf* expression in mammals. In conclusion, estrogen-responsive gene transcripts were predicted to be expressed in various tissues according to knowledge-based tissue enrichment; many of these tissues are in concordance with the ones that have been identified by transgenic estrogen reporter fish [17,27], but some of them are novel E2 target tissues for zebrafish, such as pancreas. 

### E2 regulates similar biological processes during early zebrafish development

Although the estrogen-responsive genes during early zebrafish development were expressed in a time-dependent manner, the biological functional processes were fairly similar across all time points. Analysis by GO-term biological processes enrichment for E2 regulated genes identified metabolic processes, regulation of transcription, transport, signal transduction, phosphorylation, development and immune response to be significantly enriched at all time points (Table 1). More subcategories to these main categories that were enriched also overlapped in all the time points (Tables S8-S12). However, a few categories were not significantly enriched across all time points; apoptosis and cell proliferation categories were enriched only at 3 and 4 dpf, and the response to chemical stimulus category at 2-4 dpf. 

In line with the liver being one of the major tissue targets for estrogenic signaling, E2-target genes were enriched in the metabolism category. The genes in this category encode both metabolic enzymes in the steroid and hormone pathways, as well as enzymes involved in lipid, nucleic acid, carbohydrate, protein, xenobiotic and energy reserve metabolism (Table S8). The transcripts of the major estrogen-metabolizing genes *CYP19A1*, encoding aromatase B (zebrafish homologue *cyp19a1b*), sulfotransferase *SULT1E1* (zebrafish homologue *sult2st3*) and hydroxysteroid (17β) dehydrogenase *HSD17B* (zebrafish homologue *hsd17b*) were all differentially expressed in our study; expression of *cyp19a1b and sult2st3* were up-regulated while *hsd17b* was down-regulated by E2 treatment. For lipid metabolism, *lipc* (encoding hepatic lipase) was down-regulated, while apolipoprotein A-I (encoded by *apoa*), which is the major protein component of high density lipoprotein (HDL) in plasma, was up-regulated by E2 treatment. Studies of humans and primates confirm the regulation of LIPC and APOA-1 by E2. First, E2 is known to repress the transcription expression of *LIPC* in humans, which plays an important role in lowering the plasma level of HDL [45,46], a function requiring *ESR1* [45]. Second, it has been reported that hepatic *apoa1* is induced by E2 in the human hepatoma cell line HepG2 [47]. Furthermore, APOA-1 levels and production rate were shown to increase during postmenopausal estrogen replacement therapy [48]. Finally, metabolic studies on ovariectomized and hysterectomized baboons show that E2-treatment increases APOA-1 content of HDL [49]. 

Another GO biological category that was enriched in our analysis was transport, including ion transport (specifically sodium and calcium ion transport), lipid transport, protein transport, and carbohydrate transport (including glucose transport) (Tables 1 and S9). As described above, a group of solute carrier family genes were regulated by E2, including *slc2a1* (glucose transporter)*, slc8a1* (sodium/calcium exchanger)*, slc5a5* (sodium iodide symporter) *and slc34a2* (sodium phosphate transporter). Expression of *slc2a1* has been shown to be stimulated by E2 to increase glucose uptake in various tissues [50-52] and *slc34a2* mRNA expression is increased by 50% in rat intestine after E2 treatment [53]. Expression of *slc8a1* was reported to be up-regulated by E2 in hearts [54] and *slc5a5* was up-regulated by E2 treatment in mammary gland [55,56] and breast [57,58]. However, the expression of these genes was up-regulated by estrogen in these previous reports, but down-regulated in our study, suggesting that solute carrier family may play a different role during zebrafish development than in mammals. 

E2 treatment also regulated gene transcripts that were enriched in the signal transduction and transcription factor categories (Tables 1 and S10), which is in line with how E2 functions at a molecular level. E2 signaling pathways are mediated by estrogen receptors, *ESR1* and *ESR2* (or zebrafish receptors *esr1*, *esr2a* and *esr2b*), as well as the membrane bound estrogen receptor (GPER). Signaling through these receptors has been shown to crosstalk with other receptors or transcription factors. The subcategories that were enriched in our GO analysis included steroid hormone mediated signaling pathway, cell surface receptor linked signaling pathway, as well as MAPK pathway (Table S10). Expression of several receptors was differentially regulated by E2 treatment, including *growth hormone releasing hormone receptor (ghrhr*)*, retinoid X receptor* α (*rxra*)*, esr1*, *progesterone receptor (pgr*), and *nuclear receptor subfamily 0b2a,* (*nr0b2shp*), which were up-regulated by E2, and *aryl hydrocarbon receptor (ahr*)*, retinoic acid receptor* α (*rara*)*, nuclear receptor* subfamily *5a2* (*nr5a2 lrh*)*, luteinizing hormone/choriogonadotropin receptor* (*lhcgr*)*, glucagon a* (*gcg*)*, and androgen receptor(ar*), which were down-regulated. It has been extensively reported that E2 induces *pgr* expression, which is in agreement with our data. Both *esr1* and *esr2* bind to the *pgr* promoter, and *esr1* has been described to induce *pgr* expression [59,60]. However, *esr2* has been reported to reduce *pgr* expression [61] and an increase of ESR2:ESR1 ratio may suppress PGR expression and contribute to progesterone resistance [62]. AHR signaling is known to crosstalk with ER signaling. E2 has been reported to repress AHR trans-repression through binding to ESR1 [63]. Expression of RARα has been previously shown to be up-regulated by E2 in various tissues [64-66], but our data showed the opposite regulation during zebrafish development. E2 is also known to induce expression of SHP in mouse and rat liver and in human HepG2 cells [67]. Given that LRH-1 is involved in estrogen production [68], the down-regulation observed in our data may suggest a negative feedback mechanism of E2 to LRH expression. All in all, our data shows that induction of E2 signaling translates to a crosstalk with several receptors during zebrafish development. 

E2 also altered the expression of genes involved in cell proliferation and apoptosis, such as *cysteine-rich angiogenic* inducer *61* (*cyr61*), *B-cell* lymphoma *2* (*bcl2*) and *caspase 3* (*casp3*). Expression of *cyr61* has been reported to be induced by estrogen in breast cancer cells [69,70] and human myometrial explants [70], which is consistent with the up-regulation of *cyr61* expression in our study. Expression of *bcl2* was down-regulated in our study, but previous reports have shown both induction and repression of *bcl-2* expression by estrogen, potentially in a tissue specific manner [71-75]. Expression of *casp3*, a death protease activated during apoptosis, was also down-regulated during zebrafish development following E2 treatment. It has been shown that E2 at neuroprotective doses blocks *casp3* activation in the hippocampal CA1 of male gerbils [76]. In fetal neuroepithelial cells, E2 strongly inhibits the activation of *casp3* [76]. In accordance with the previous studies, expression of *cyclin-dependent kinase 5 regulatory* subunit *1a* (*cdk5*, p35) [77] and *protein tyrosine phosphatase receptor* type *C* (*ptprc*) [78] was up-regulated upon E2 treatment. 

Our data identified several phosphatase genes that are regulated by E2. Such genes include *dual specificity phosphatase 3* (*dusp3*), which has been shown to dephosphorylate and inactivate various MAPKs like ERK and JNK [79,80], and *protein phosphatase 1, regulatory* (*inhibitor*) subunit *1C* (*ppp1r1c*). Finally, the category immune response was also enriched amongst E2-regulated gene transcripts (Table 1). In our gene list, *interferon-gamma* (*ifng1-1*), a vital immunoregulatory cytokine, was up-regulated, which is in agreement with previous studies that reported an increase of IFNG secretion following estrogen treatment in mice and rats [81,82]. Both ESR1 and GPER have been shown to increase IFNG expression [83-85]. Tumor necrosis factor receptor superfamily 1a (*tnfrsf1*) was down-regulated in zebrafish embryos after E2 treatment, which is consistent with a study showing that E2 inhibits TNFR1 expression in breast adipose fibroblasts [76]. 

In conclusion, our data reveal distinct differences in the cohort of E2-responsive genes across different developmental stages in the zebrafish. Tissue enrichment analysis of E2-responsive genes correlated to tissue-specific GFP expression of *Tg*(*5xERE:GFP*) transgenic fish. However, *Tg*(*5xERE:GFP*) fish cannot mirror all E2-responsive tissues since the GFP expression is ERE driven. Our study revealed E2 responsive genes independently of whether E2 targeted *esr1, 2a* or *2b* or *gper*. The new target genes may potentially play important roles for estrogen-mediated regulation of development and some may serve as biomarkers to score for endocrine disruption. To further the knowledge of how estrogen regulates embryonic development, or the impact of perturbed estrogen signaling by exposure to estrogen disrupting compounds, it will be necessary to map estrogen receptor type specific responses through selective estrogen modulators and knockdown/knockout of Esr1, 2a, 2b or Gper. 

## Supporting Information

Figure S1 **Principle components analysis of microarray samples.** (A) Untreated samples (vehicle only 0.1% DMSO). (B) Samples treated with E2.
(TIF)

Figure S2 **Uptake of E2 in embryos at different time points.** Thirty wild type zebrafish embryos were pooled and treated with 1 μM E2 (in 0.1% DMSO) from 3 hpf. The media was collected every 24 hours at 1, 2, 3 and 4 dpf, and the amount of E2 remaining in the media was analyzed by UV-HPLC. The data is presented as the percentage of E2 remaining in the water relative to media incubated with E2 but without fish. Statistics were done using Student’s t-test. *P<0.05; **P<0.01; ***P<0.005; ns, not significant.
(TIF)

Figure S3 **Correlation analysis of gene expression data from RT-qPCR and microarray experiments.** The relative fold change values of each gene at each time point from Figure 4 were used for the correlation analysis. The microarray data (Y axis) were plotted against the RT-qPCR data (X axis).
(TIF)

Figure S4 **18S rRNA expression of 1-4 dpf embryos and adult fish upon E2 treatment (relative to DMSO treatment).**
(TIF)

Figure S5 **E2 up-regulates expression of *vtg4* in the liver of 4 dpf DZ zebrafish embryos.** Whole-mount ISH was performed with anti-sense *vtg4* RNA probes on 4 dpf E2-treated embryos (A) and DMSO-treated embryos (B). ISH of sense *vtg4* RNA probes on E2-treated embryos (C) and DMSO-treated embryos were performed as controls. Lateral view; anterior to the left. *Arrow* (*red*) indicates expression location of *vtg4* in the liver. *Scale* *bars*, 200 μm.
(TIF)

Figure S6 **Maternal effect of *Tg*(*5xERE:GFP*) transgenic fish at 5 hpf in the absence of E2.** (A, B) *Tg*(*5xERE:GFP*) transgenic fish embryos. (C, D) Embryos from cross of female *Tg*(*5xERE:GFP*) transgenic fish and male wild type DZ fish. (E, F) Embryos from cross of male *Tg*(*5xERE:GFP*) transgenic fish and female wild type DZ fish. (G, H) Wild type DZ fish embryos. A, C, E and G, bright-field images; B, D, F and H corresponding GFP fluorescence images; *Scale* *bars*, 500 μm.
(TIF)

Figure S7 **Endocrine pancreas is a novel E2 responsive tissue in embryonic zebrafish.** Double transgenic *Tg*(*5xERE:GFP*)*/Tg*(*ins:mCherry*) embryos (4dpf) showing co-localization of GFP and mCherry signals in pancreatic islets upon E2 treatment. (A) GFP fluorescence image; (B) mCherry fluorescence image; (C) merged image of GFP and mCherry; (D) merged image of bright field (BF), GFP and mCherry. *Arrows* indicate the liver; *arrowheads* indicate the pancreatic islets. Dorsal view; anterior to the left. *Scale* *bars*, 100 μm.
(TIF)

File S1 **List of genes with E2-altered expression at 1, 2, 3 and 4 dpf.**
(XLSX)

Table S1 Primer sequences used for the RT-qPCR validation of estrogen responsive genes from the microarray.
(DOCX)

Table S2 Number of differentially expressed genes at different developmental stages.
(DOCX)

Table S3 Top 15 up- and down-regulated transcripts at 1 dpf upon E2 treatment (E2 vs control).
(DOCX)

Table S4 Top 15 up- and down-regulated transcripts at 2 dpf upon E2 treatment (E2 vs control).
(DOCX)

Table S5 Top 15 up- and down-regulated transcripts at 3 dpf upon E2 treatment (E2 vs control).
(DOCX)

Table S6 Top 15 up- and down-regulated transcripts at 4 dpf upon E2 treatment (E2 vs control).
(DOCX)

Table S7 Common estrogen responsive genes at 1 dpf, 2 dpf, 3 dpf and 4 dpf upon E2 treatment.
(DOCX)

Table S8 GO terms sub-grouped into the metabolic process category (in italics).
(DOCX)

Table S9 GO terms sub-grouped into the transport category (in italics).
(DOCX)

Table S10 GO terms sub-grouped into the signaling pathways category (in italics).
(DOCX)

Table S11 GO terms sub-grouped into the multicellular organismal development category (in italics).
(DOCX)

Table S12 GO terms sub-grouped into the response to chemical stimulus category (in italics).
(DOCX)

Table S13 Number of genes enriched by the NIH-DAVID tissue enrichment platform.
(DOCX)
