# Supplementary figures and images for: Identification of Estrogen Target Genes during Zebrafish Embryonic Development through Transcriptomic Analysis
Source: PLoS One. 2013 Nov 6;8(11):e79020. doi: 10.1371/journal.pone.0079020 (PMC3819264; doi:10.1371/journal.pone.0079020)

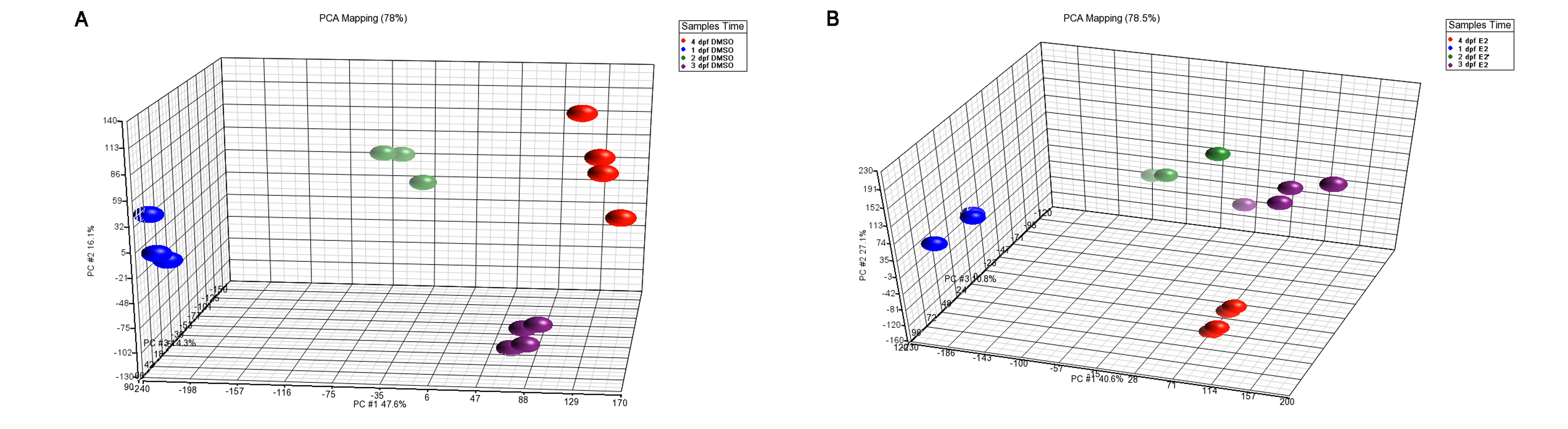

Supplement: Figure S1 — Principle components analysis of microarray samples. (A) Untreated samples (vehicle only 0.1% DMSO). (B) Samples treated with E2. (TIF) [file pone.0079020.s001.tif]

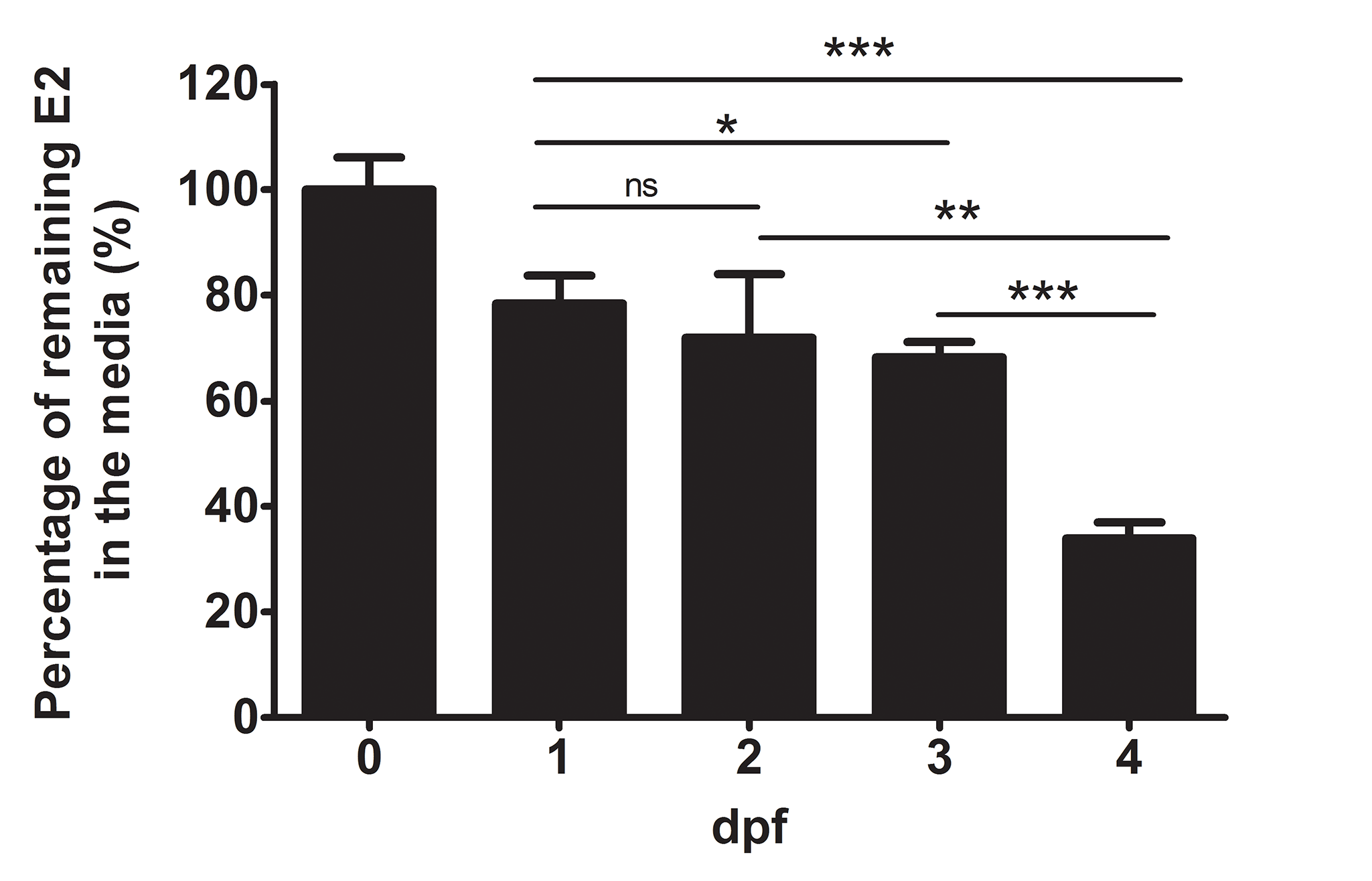

Supplement: Figure S2 — Uptake of E2 in embryos at different time points. Thirty wild type zebrafish embryos were pooled and treated with 1 μM E2 (in 0.1% DMSO) from 3 hpf. The media was collected every 24 hours at 1, 2, 3 and 4 dpf, and the amount of E2 remaining in the media was analyzed by UV-HPLC. The data is presented as the percentage of E2 remaining in the water relative to media incubated with E2 but without fish. Statistics were done using Student’s t-test. *P<0.05; **P<0.01; ***P<0.005; ns, not significant. (TIF) [file pone.0079020.s002.tif]

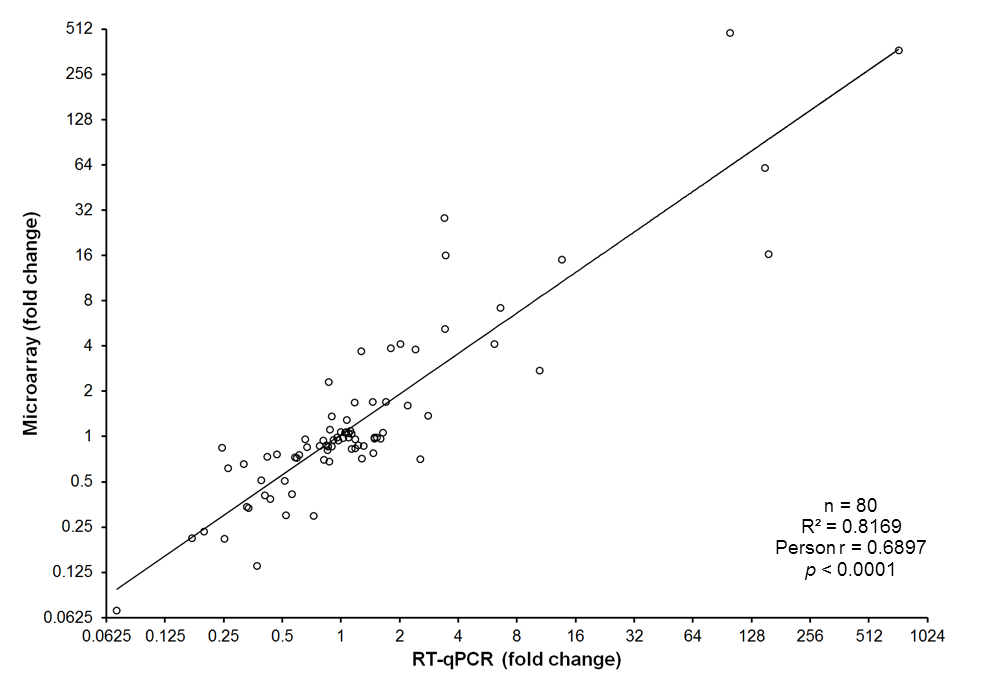

Supplement: Figure S3 — Correlation analysis of gene expression data from RT-qPCR and microarray experiments. The relative fold change values of each gene at each time point from Figure 4 were used for the correlation analysis. The microarray data (Y axis) were plotted against the RT-qPCR data (X axis). (TIF) [file pone.0079020.s003.tif]

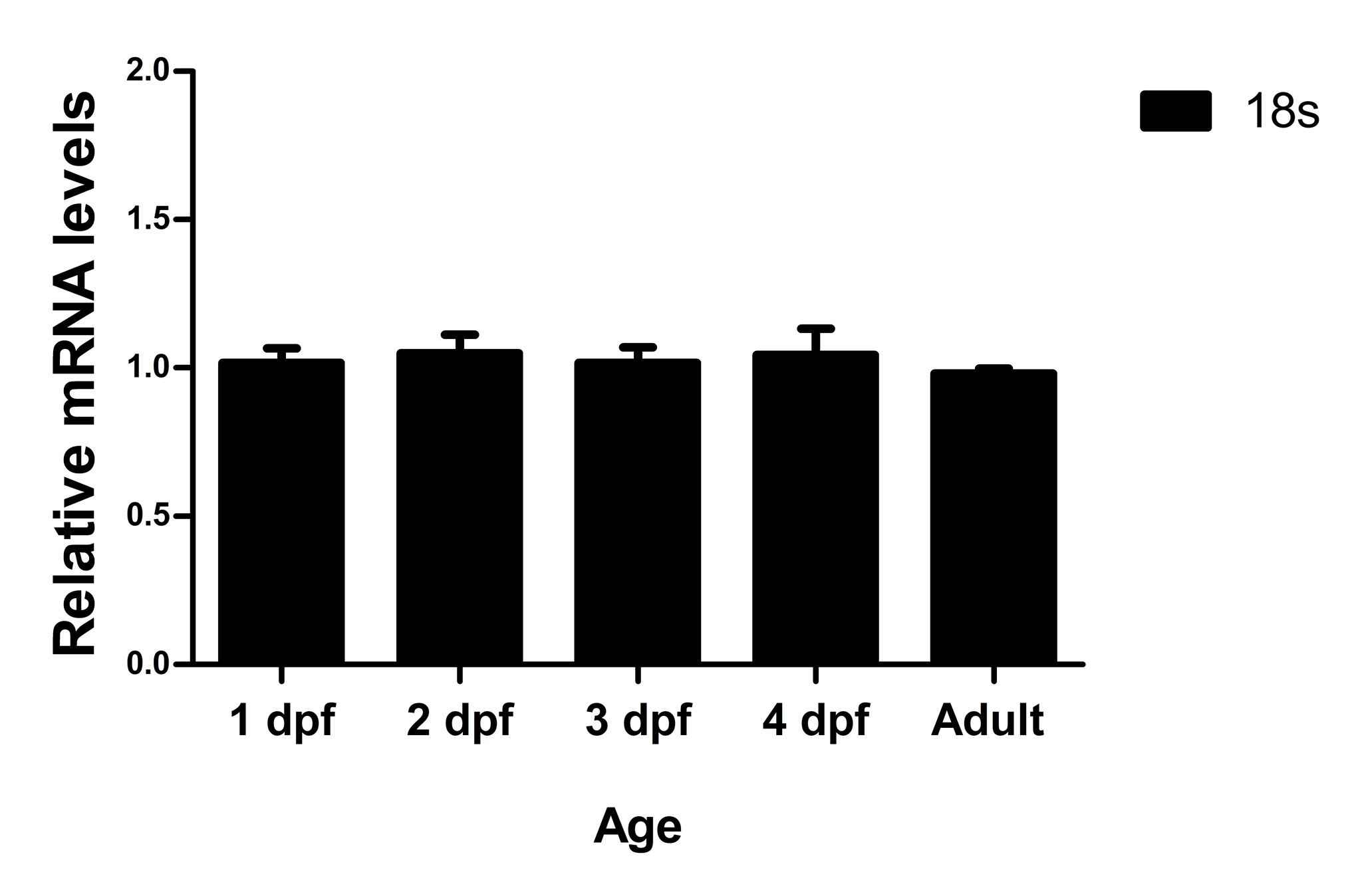

Supplement: Figure S4 — 18S rRNA expression of 1-4 dpf embryos and adult fish upon E2 treatment (relative to DMSO treatment). (TIF) [file pone.0079020.s004.tif]

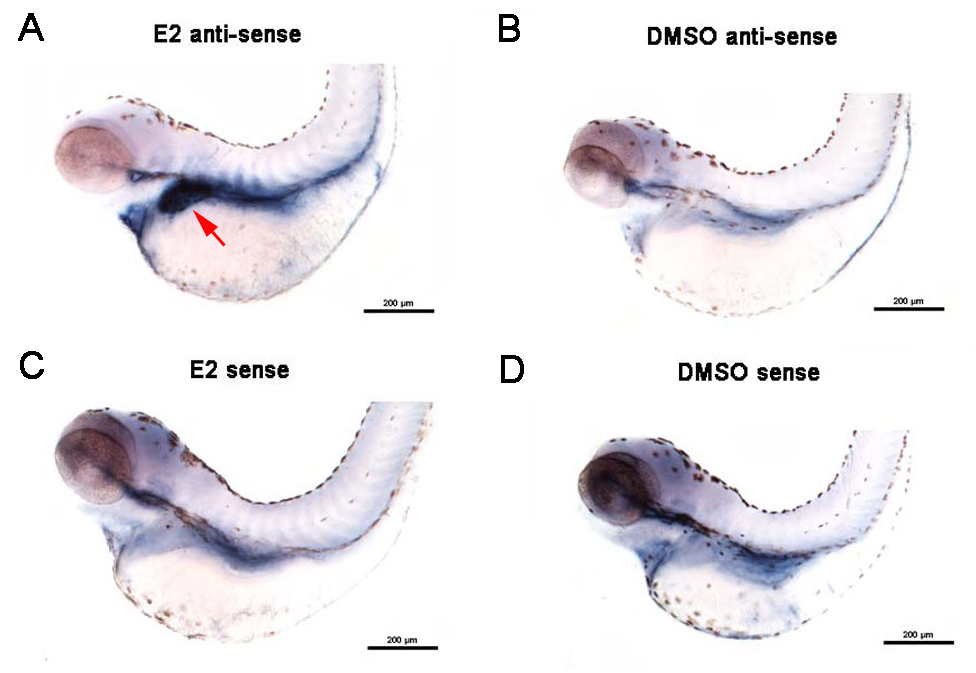

Supplement: Figure S5 — E2 up-regulates expression of vtg4 in the liver of 4 dpf DZ zebrafish embryos. Whole-mount ISH was performed with anti-sense vtg4 RNA probes on 4 dpf E2-treated embryos (A) and DMSO-treated embryos (B). ISH of sense vtg4 RNA probes on E2-treated embryos (C) and DMSO-treated embryos were performed as controls. Lateral view; anterior to the left. Arrow (red) indicates expression location of vtg4 in the liver. Scale bars, 200 μm. (TIF) [file pone.0079020.s005.tif]

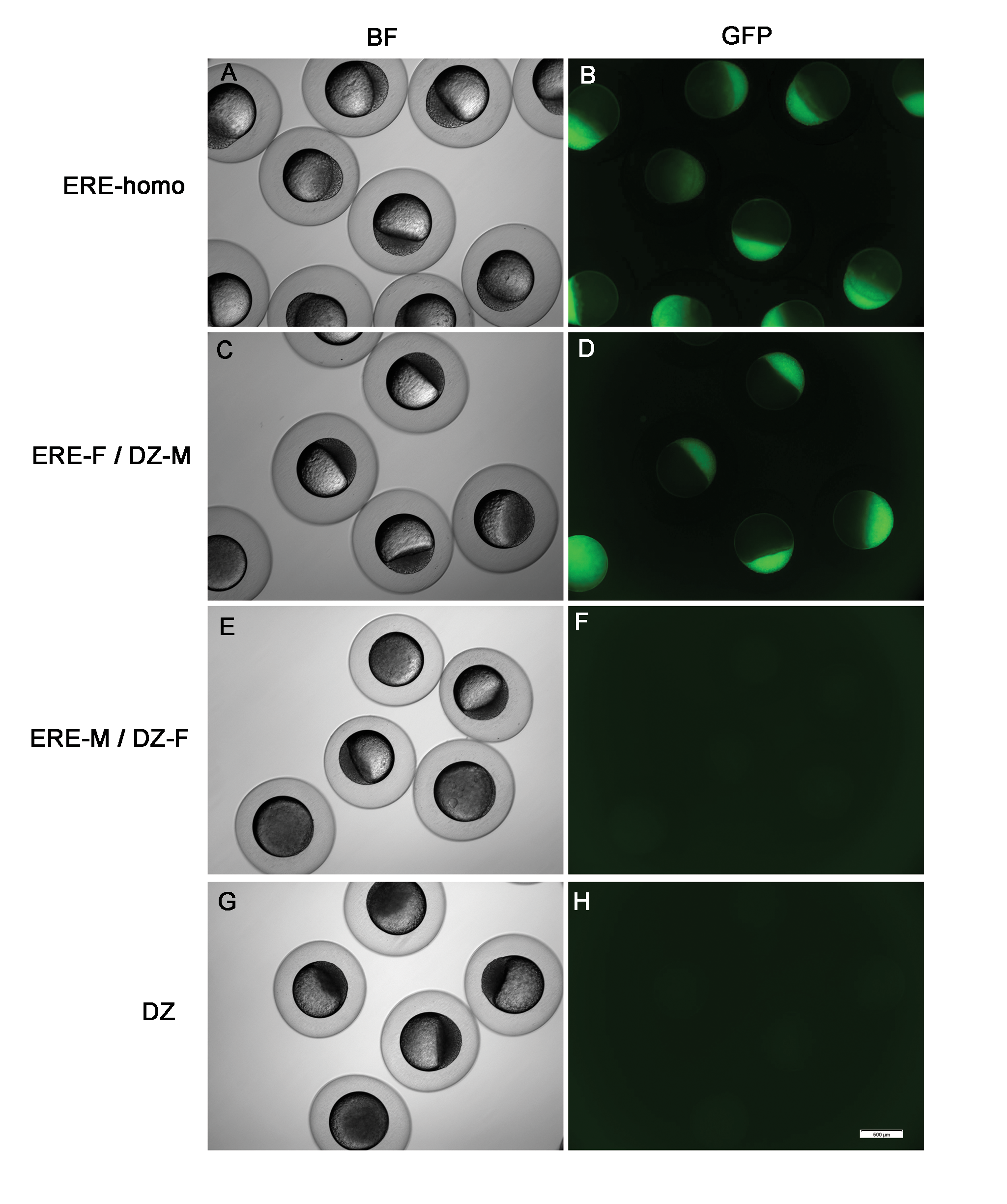

Supplement: Figure S6 — Maternal effect of Tg(5xERE:GFP) transgenic fish at 5 hpf in the absence of E2. (A, B) Tg(5xERE:GFP) transgenic fish embryos. (C, D) Embryos from cross of female Tg(5xERE:GFP) transgenic fish and male wild type DZ fish. (E, F) Embryos from cross of male Tg(5xERE:GFP) transgenic fish and female wild type DZ fish. (G, H) Wild type DZ fish embryos. A, C, E and G, bright-field images; B, D, F and H corresponding GFP fluorescence images; Scale bars, 500 μm. (TIF) [file pone.0079020.s006.tif]

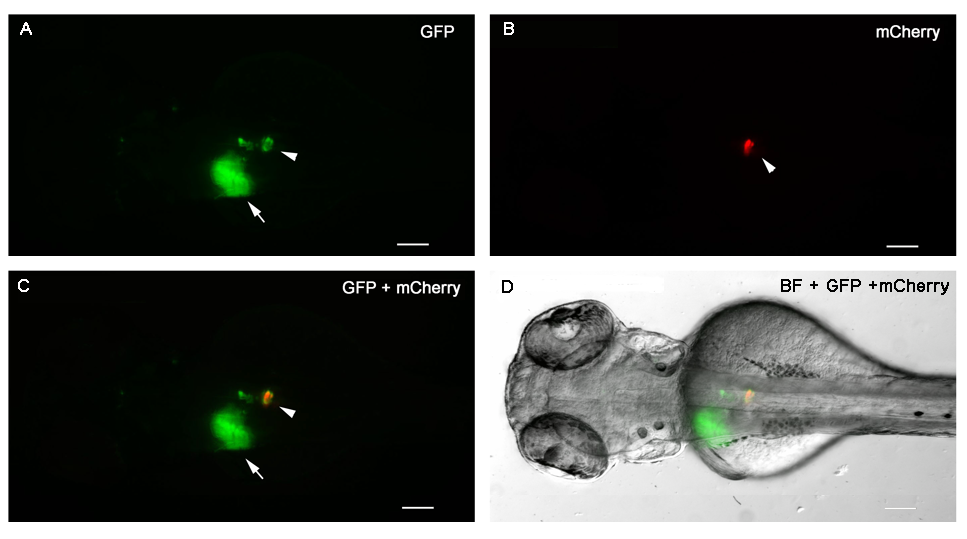

Supplement: Figure S7 — Endocrine pancreas is a novel E2 responsive tissue in embryonic zebrafish. Double transgenic Tg(5xERE:GFP)/Tg(ins:mCherry) embryos (4dpf) showing co-localization of GFP and mCherry signals in pancreatic islets upon E2 treatment. (A) GFP fluorescence image; (B) mCherry fluorescence image; (C) merged image of GFP and mCherry; (D) merged image of bright field (BF), GFP and mCherry. Arrows indicate the liver; arrowheads indicate the pancreatic islets. Dorsal view; anterior to the left. Scale bars, 100 μm. (TIF) [file pone.0079020.s007.tif]
