# Supplementary material for: Identification of Estrogen Target Genes during Zebrafish Embryonic Development through Transcriptomic Analysis
Source: PLoS One. 2013 Nov 6;8(11):e79020. doi: 10.1371/journal.pone.0079020 (PMC3819264; doi:10.1371/journal.pone.0079020)
Supplement: Table S1 — Primer sequences used for the RT-qPCR validation of estrogen responsive genes from the microarray. (DOCX) [file pone.0079020.s009.docx]

Table S1. Primer sequences used for the RT-qPCR validation of estrogen responsive genes from the microarray

| **Genes** | **Forward primer sequences (5’-3’)** | **Reverse primer sequences (5’-3’)** | **Genebank**  **accession no.** |
| --- | --- | --- | --- |
| **Reference gene (*18s* ribosomal RNA)** | | | |
| *18s* ^(1)^* | TCGCTAGTTGGCATCGTTTATG | CGGAGGTTCGAAGACGATCA | BX296557 |
| **Up-regulated genes** | | | |
| *esr1^#(2)^* | CAGGACCAGCCCGATTCC | TTAGGGTACATGGGTGAGAGTTTG | NM_152959 |
| *vtg1^$(3)^* | ACTACCAACTGGCTGCTTAC | ACCATCGGCACAGATCTTC | NM_170767 |
| *vtg3^$(3)^* | CAGATGGCTTTATCGGCGTGAC | CACGGCAGGCCCATTGAAAC | AF254638 |
| *vtg5^%(4)^* | CCAAAAATTGTCACCACTTATGCT | CTTCATTCCTCCATGATATGCTTA | BC142783.1 |
| *cyp19a1b* | AAAGAGTTACTAATAAAGATCCACCGGTAT | TCCACAAGCTTTCCCATTTCA | AF226619 |
| *f13a1a* | GCCCGTATTGCTTTTGCC | GTGGGCCTGCTTGTTTTCTG | [NM_001076711.1](http://www.ncbi.nlm.nih.gov/nuccore/NM_001076711.1) |
| *eif4e1b* | GACACGGTCGAGGACTTCTG | GTGTGCCTATGTTGCTTGGC | [NM_131454.1](http://www.ncbi.nlm.nih.gov/nuccore/NM_131454.1) |
| *cyp11a1* | GAGGCCTCAGGAATGTCCAC | GGTCCACGCGTCTACATTGA | [NM_152953.2](http://www.ncbi.nlm.nih.gov/nuccore/NM_152953.2) |
| *dazl* | TGTTCGTCGGCGGTATTGAT | TGACACTGACCGAGAACTTCG | [NM_131524.1](http://www.ncbi.nlm.nih.gov/nuccore/NM_131524.1) |
| *zp3* | TCCAGCCAGTGGGTCTGACTA | CCAACAATTGCACCATCAGTCC | [NW_003040435.2](http://www.ncbi.nlm.nih.gov/nucleotide/312123451?report=gbwithparts) |
| *cpn1* | TGAGGGCTCTTTTTGCCGTC | GACGTACTTGAACTCCGGCT | [NW_001877244.3](http://www.ncbi.nlm.nih.gov/nucleotide/312124172?report=gbwithparts) |
| **Down-regulated genes** | | | |
| *agxtb* | TCATCAGCTGGTTTGGTGCACCC | GAGCAGCTCCCAATGATGCCAC | NM_213162.1 |
| *pnp4b* | CGCATGCTGCACATCTTGGGGA | TGAGCGAGAGACCCATGACCCG | NM_205643.1 |
| *hpx* | CTCATAAAGGCAAACCTGGTG | TGGACAGCTCAGCCTTGCCA | NM_001111147 |
| *fabp10a* | AGCTGGTCTGCAGAACTGACCGA | TGGTGGTTCCTCCGACTGTCAGC | NM_152960.1 |
| *fkbp5* | TCCACGAACCAGTGCCCGACT | AGTGGACGAACACCCTGTCCC | NM_213149.1 |
| *klf9* | CACGGAAGCGCGACCGACTG | GAAAGGGCCTCTCACCGGTGTG | NM_001128729.1 |
| *zgc:110053* | GCCAACTCGCCACAGCCAGAC | CGAACTCCGGGCTCACACCGA | NM_001020562.1 |
| *nxf1* | GGCCTCGGAAGAGCAGAAATCTTC | TCATTGAGGTGACCCAGTGCTGA | XM_001343386.3 |
| *zgc:92590* | GAGCCCAGTGTGAGGGTGCGTA | CCGCCAGAATCTCCCTGACATGC | NM_001007054.1 |
| *f2* | ACTGTCAGGAGGGAGACCTG | CGCTCTCCACAGTCTAGCTC | [NM_213390.1](http://www.ncbi.nlm.nih.gov/nuccore/NM_213390.1) |
| *sult1st3* | TTCACTACTTCACTGACAACTGG | CAGCTCTGTTCCGAATGGTATC | [NM_183348.2](http://www.ncbi.nlm.nih.gov/nucleotide/56118729?report=gbwithparts) |
| **Non-changed genes** | | | |
| *dlgap* | GAAGCTCCCGCCGCCAGTAC | GCGCGTTTGGCTGCCATGAG | XM_680713.4 |
| *rbp2a* | ACTGTTAAGACCCTGGTAAAGTGGG | GAATCTCCAAGTGAAGCAAGTCTCC |  |
| *esr2b^#(2)^* | CGCTCGGCATGGACAAC | CCCATGCGGTGGAGAGTAAT | AAH86848 |
| *esr2a^#(2)^* | CTCACAGCACGGACCCTAAAC | GGTTGTCCATCCTCCCGAAAC | NM_001045184 |

References:

* (1) McCurley A and Callard G. (2008). Characterization of housekeeping genes in zebrafish: male-female differences and effects of tissue type, developmental stage and chemical treatment. BMC Molecular Biology. 9(102): 1471-2199

# (2) Chandrasekar G, Archer A, Gustafsson JA, Lendahl MA. (2010).Levels of 17b-Estradiol Receptors Expressed in Embryonic and Adult Zebrafish Following In Vivo Treatment of Natural or Synthetic Ligands. PLoS ONE. 5 (3): e9678.

$ (3) Meng X, Bartholomew C and Craft JA (2010). Differential expression of vitellogenin and oestrogen receptor genes in the liver of zebrafish*, Danio Rerio.* Anal Bioanal Chem: 396: 625-630

% (4) Sawyer S, Gerstner K and Callard G. (2006). Real-time PCR analysis of cytochrome P450 aromatase expression in zebrafish: Gene specific tissue distribution, sex differences, developmental programming, and estrogen regulation. General and Comparative Endocrinology 147: 108–117.
