# Supplementary material for: Identification of Estrogen Target Genes during Zebrafish Embryonic Development through Transcriptomic Analysis
Source: PLoS One. 2013 Nov 6;8(11):e79020. doi: 10.1371/journal.pone.0079020 (PMC3819264; doi:10.1371/journal.pone.0079020)
Supplement: Table S2 — Number of differentially expressed genes at different developmental stages. (DOCX) [file pone.0079020.s010.docx]

Table S2. Number of differentially expressed genes at different developmental stages

|  | ***p* ≤ 0.005** | ***p* ≤ 0.01** | ***p* ≤ 0.01(Fold change ≥ \|±1.4\|)** |
| --- | --- | --- | --- |
| **1 dpf** | | | |
| Total probes | 140 | 323 | 298 |
| Probes with gene symbols | 74 | 156 | 140 |
| Gene number | 70 | 151 | 136 |
| **2 dpf** | | | |
| Total probes | 158 | 376 | 219 |
| Probes with gene symbols | 114 | 249 | 116 |
| Gene number | 101 | 230 | 104 |
| **3 dpf** | | | |
| Total probes | 418 | 1277 | 1016 |
| Probes with gene symbols | 301 | 853 | 625 |
| Gene number | 267 | 778 | 576 |
| **4 dpf** | | | |
| Total probes | 368 | 745 | 444 |
| Probes with gene symbols | 228 | 461 | 227 |
| Gene number | 208 | 425 | 204 |
