# Supplementary material for: Identification of Estrogen Target Genes during Zebrafish Embryonic Development through Transcriptomic Analysis
Source: PLoS One. 2013 Nov 6;8(11):e79020. doi: 10.1371/journal.pone.0079020 (PMC3819264; doi:10.1371/journal.pone.0079020)
Supplement: Table S3 — Top 15 up- and down-regulated transcripts at 1 dpf upon E2 treatment (E2 vs control). (DOCX) [file pone.0079020.s011.docx]

| **Gene Symbol** | **Human homologue** | ***p*-value** | **Fold-Change** | **Genbank Accession** |
| --- | --- | --- | --- | --- |
| **Up-regulated genes** | | | | |
| *vtg3* |  | 1.23E-04 | 6.21 | AF254638 |
| *dcaf13* | *DCAF13* | 7.60E-05 | 4.73 | NM_200129 |
| *vtg1* |  | 8.65E-04 | 3.82 | NM_001044897 |
| *taar19p* | *TAAR6* | 2.42E-03 | 3.58 | NM_001199914 |
| *col10a1* | *COL10A1* | 7.61E-03 | 3.39 | NM_001083827 |
| *ptprc* | *PTPRC* | 4.78E-03 | 2.71 | CK127788 |
| *zgc:153505* | *DPYD* | 4.53E-03 | 2.70 | NM_001077308 |
| *ppp1r1c* | *PPP1R1C* | 7.26E-05 | 2.59 | NM_001002538.1 |
| *dusp3* | *DUSP3* | 1.13E-03 | 2.50 | NM_001044307 |
| *mettl16* | *METTL16* | 1.22E-02 | 2.43 | BC131878 |
| *ugt5c3* |  | 4.79E-03 | 2.38 | NM_001128714 |
| *rh50* |  | 6.42E-04 | 2.30 | NM_131547 |
| *c1qtnf2* | *C1QTNF2* | 3.26E-03 | 2.22 | XM_695699 |
| *hoxd12a* | *HOXD12* | 5.56E-03 | 2.20 | NM_001126486 |
| *igsf21b* | *IGSF21* | 1.39E-03 | 2.18 | NM_001110473 |
| **Down-regulated genes** | | | | |
| *znf644* | *ZNF644* | 9.11E-03 | -5.32 | CT700944 |
| *nitr3d* |  | 1.21E-03 | -4.43 | NM_198355 |
| *ddc* | *DDC* | 3.07E-03 | -3.94 | NM_213342 |
| *gria4a* | *GRIA4* | 1.05E-02 | -3.82 | NM_214806 |
| *opn1lw2* | *OPN1LW* | 5.92E-04 | -3.57 | NM_001002443 |
| *prnp* | *PRNP* | 1.16E-02 | -3.49 | NM_205586 |
| *zgc:193725* |  | 5.67E-03 | -3.40 | EH551598 |
| *npy7r* |  | 9.50E-03 | -3.29 | NM_001007218 |
| *slc7a11* | *SLC7A1* | 1.40E-02 | -3.13 | XM_001919391 |
| *zgc:112320* |  | 4.79E-03 | -3.13 | NM_001159826 |
| *nr1h5* | *NR1H5* | 8.09E-03 | -3.05 | NM_001123241 |
| *dpp6b* | *DPP6* | 3.21E-03 | -3.04 | NM_001115122 |
| *hmgcll1* | *HMGCLL1* | 1.76E-03 | -2.95 | NM_001110400 |
| *sulf2l* |  | 3.38E-04 | -2.71 | NM_001003833 |
| *grid2* | *GRID2* | 8.27E-05 | -2.59 | NM_001004123 |

Table S3. Top 15 up- and down-regulated transcripts at 1 dpf upon E2 treatment (E2 vs control)
