# Supplementary material for: Identification of Estrogen Target Genes during Zebrafish Embryonic Development through Transcriptomic Analysis
Source: PLoS One. 2013 Nov 6;8(11):e79020. doi: 10.1371/journal.pone.0079020 (PMC3819264; doi:10.1371/journal.pone.0079020)
Supplement: Table S4 — Top 15 up- and down-regulated transcripts at 2 dpf upon E2 treatment (E2 vs control). (DOCX) [file pone.0079020.s012.docx]

Table S4. Top 15 up- and down-regulated transcripts at 2 dpf upon E2 treatment (E2 vs control)

| **Gene Symbol** | **Human homologue** | ***p*-value** | **Fold-Change** | **Genbank Accession** |
| --- | --- | --- | --- | --- |
| **Up-regulated genes** | | | | |
| *f13a1a* | *F13A1* | 1.64E-09 | 28.39 | NM_001076711 |
| *cyp19a1b* | *CYP19A1* | 2.65E-04 | 7.58 | NM_131642 |
| *vtg1* |  | 3.28E-05 | 4.50 | NM_001044897.2 |
| *dnah10* | *DNAH* | 4.64E-03 | 4.02 | XM_693415 |
| *vipr1* | *VIPR1* | 1.10E-03 | 3.68 | NM_001013353 |
| *amh* | *AMH* | 2.08E-05 | 2.88 | NM_001007779 |
| *asb15b* | *ASB15* | 1.49E-02 | 2.80 | NM_001039890 |
| *desmb* | *DES* | 6.35E-03 | 2.41 | NM_001077452 |
| *coch* | *COCH* | 2.54E-03 | 2.33 | NM_001003823 |
| *or110-2* | *OR1Q1* | 1.05E-02 | 2.01 | NM_001128411.1 |
| *map1s* | *MAP1S* | 3.68E-03 | 1.91 | XM_688813 |
| *mxtx1* | *DUXA* | 7.99E-03 | 1.73 | NM_131560 |
| [rxraa](http://useast.ensembl.org/Danio_rerio/geneview?gene=rxraa) | *RXRA* | 1.17E-03 | 1.68 | NM_001161551.1 |
| *zgc:172270* |  | 1.35E-02 | 1.61 | NM_001114568 |
| *nr0b2a* | *NR0B2* | 4.58E-03 | 1.53 | NM_001256191 |
| **Down-regulated genes** | | | | |
| *fkbp5* | *FKBP5* | 3.40E-08 | -15.81 | NM_213149 |
| *klf9* | *KLF9* | 1.87E-05 | -4.28 | NM_001128729 |
| *pglyrp2* | *PGLYRP2* | 4.66E-03 | -3.62 | NM_001045166 |
| *fabp10a* |  | 5.83E-04 | -3.37 | NM_152960 |
| *zgc:162180* |  | 2.86E-03 | -2.94 | NM_001089446 |
| *pnp4b* |  | 2.96E-04 | -2.90 | NM_205643 |
| *hpx* | *HPX* | 4.95E-04 | -2.86 | NM_001111147 |
| *ddc* | *DDC* | 6.01E-04 | -2.80 | NM_213342 |
| *epd* |  | 5.48E-05 | -2.71 | NM_131005 |
| *kcnh6* | *KCNH6* | 1.16E-02 | -2.61 | XM_693502 |
| *rhcga* | *RHCG* | 3.18E-03 | -2.59 | NM_001089577 |
| *unc13d* | *UNC13C* | 8.50E-04 | -2.57 | CT598129 |
| *cxcr3.1* | *CXCR3* | 6.34E-03 | -2.55 | NM_001089430 |
| *nr1d2a* |  | 1.99E-03 | -2.50 | NM_001130592 |
| *ctsbb* | *CTSB* | 8.66E-05 | -2.40 | NM_001110478 |
