# Supplementary material for: Identification of Estrogen Target Genes during Zebrafish Embryonic Development through Transcriptomic Analysis
Source: PLoS One. 2013 Nov 6;8(11):e79020. doi: 10.1371/journal.pone.0079020 (PMC3819264; doi:10.1371/journal.pone.0079020)
Supplement: Table S5 — Top 15 up- and down-regulated transcripts at 3 dpf upon E2 treatment (E2 vs control). (DOCX) [file pone.0079020.s013.docx]

Table S5. Top 15 up- and down-regulated transcripts at 3 dpf upon E2 treatment (E2 vs control)

| **Gene Symbol** | **Human homologue** | ***p*-value** | **Fold-Change** | **Genbank Accession** |
| --- | --- | --- | --- | --- |
| **Up-regulated genes** | | | | |
| *cyp19a1b* | *CYP19A1* | 1.92E-08 | 35.10 | NM_131642 |
| *vtg1* |  | 2.00E-08 | 7.91 | NM_001044897 |
| *batf* |  | 1.07E-03 | 6.06 | NM_001045059 |
| *c14orf135* | *C14ORF135* | 1.22E-02 | 5.70 | CT698302 |
| *cd74b* | *CD74* | 1.51E-03 | 5.60 | NM_131372 |
| *cyp11a1* | *CYP11A1* | 3.39E-03 | 5.20 | NM_152953 |
| *dazl* | *DAZL* | 1.32E-02 | 4.97 | NM_131524 |
| *hoxb9a* | *HOXB9* | 1.32E-02 | 4.74 | NM_131121 |
| *ccdc37* | *CCDC37* | 5.17E-03 | 4.51 | NM_001076760 |
| *morc3b* | *MORC3* | 1.12E-02 | 4.51 | NM_001003579 |
| *dcst2* | *DCST2* | 7.94E-03 | 4.36 | XM_679060 |
| *ubn2* | *UBN2* | 2.95E-03 | 4.12 | NM_001104940 |
| *h1m* | *H1FOO* | 7.88E-03 | 4.09 | NM_183071 |
| *exosc6* | *EXOSC6* | 1.14E-02 | 3.98 | BC065602 |
| *f13a1a* | *F13A1* | 9.44E-05 | 3.88 | NM_001076711 |
| **Down-regulated genes** | | | | |
| *fabp10a* |  | 2.23E-07 | -7.22 | NM_152960 |
| *fabp6* | *FABP6* | 2.01E-04 | -7.11 | NM_001002076 |
| *fkbp5* | *FKBP5* | 5.42E-06 | -5.44 | NM_213149 |
| *zgc:193725* |  | 2.39E-04 | -4.61 | EH551598 |
| *hpx* | *HPX* | 3.45E-04 | -4.46 | BC056563 |
| *dupd1* | *DUPD1* | 1.78E-03 | -4.28 | NM_001039837 |
| *itln3* | *ITLN2* | 1.53E-03 | -4.25 | NM_001159584 |
| *ca4b* | *CA4* | 6.62E-03 | -4.23 | NM_001166211 |
| *serpinf2* | *SERPINF2* | 3.53E-05 | -4.10 | XM_683705 |
| *rhcga* | *RHCG* | 1.66E-05 | -4.00 | NM_001089577 |
| *rhcgb* | *RHCG* | 5.79E-03 | -3.97 | NM_207082 |
| *slc6a19b* | *SLC6A19* | 6.03E-03 | -3.70 | NM_199736 |
| *tdo2a* | *TDO2* | 1.25E-02 | -3.69 | NM_001102616 |
| *c3b* | *C3* | 1.21E-02 | -3.61 | NM_131243 |
| *shbg* | *SHBG* | 2.75E-04 | -3.50 | NM_001007151 |
