# Supplementary material for: Identification of Estrogen Target Genes during Zebrafish Embryonic Development through Transcriptomic Analysis
Source: PLoS One. 2013 Nov 6;8(11):e79020. doi: 10.1371/journal.pone.0079020 (PMC3819264; doi:10.1371/journal.pone.0079020)
Supplement: Table S6 — Top 15 up- and down-regulated transcripts at 4 dpf upon E2 treatment (E2 vs control). (DOCX) [file pone.0079020.s014.docx]

Table S6. Top 15 up- and down-regulated transcripts at 4 dpf upon E2 treatment (E2 vs control)

| **Gene Symbol** | **Human homologue** | ***p*-value** | **Fold-Change** | **Genbank Accession** |
| --- | --- | --- | --- | --- |
| **Up-regulated genes** | | | | |
| *vtg4* |  | 1.08E-15 | 1218.93 | NM_001045294 |
| *vtg3* |  | 1.88E-14 | 646.00 | AF254638 |
| *vtg1* |  | 3.12E-17 | 522.25 | NM_001044897 |
| *cyp19a1b* | *CYP19A1* | 2.28E-10 | 102.00 | NM_131642 |
| *vtg2* |  | 1.67E-08 | 44.03 | NM_001044913 |
| *f13a1a* | *F13A1* | 3.35E-09 | 16.08 | NM_001076711 |
| *vtg5* |  | 4.86E-15 | 14.96 | NM_001025189 |
| *wipf2* | *WIPF2* | 8.35E-03 | 9.73 | NM_001002165 |
| *lhbeta1* |  | 1.31E-03 | 5.05 | NM_205623 |
| *zgc:153138* |  | 2.62E-03 | 4.72 | NM_001076641 |
| *esr1* | *ESR1* | 1.94E-04 | 4.13 | NM_152959 |
| *wdhd1* | *WDHD1* | 6.08E-04 | 3.60 | NM_001002726 |
| *prop1* | *PROP1* | 4.55E-03 | 3.43 | NM_001177461 |
| *amh* | *AMH* | 1.40E-06 | 3.07 | NM_001007779 |
| *tmem232* | *TMEM232* | 5.48E-04 | 2.71 | NM_001100053 |
| **Down-regulated genes** | | | | |
| *pfkfb4* | *PFKFB4* | 4.34E-04 | -8.97 | XM_002666441 |
| *upp2* | *UPP2* | 3.87E-04 | -6.62 | NM_200144 |
| *ahsg* | *AHSG* | 7.52E-11 | -5.61 | NM_001100029 |
| *cbln8* |  | 3.88E-06 | -5.56 | NM_001110109 |
| *zgc:92590* |  | 4.68E-04 | -4.76 | NM_001007054 |
| *hpx* | *HPX* | 3.40E-05 | -4.32 | NM_001111147 |
| *il11b* | *IL11* | 1.35E-02 | -3.68 | XM_003200549 |
| *il34* | *IL34* | 1.17E-02 | -3.63 | NM_001082955 |
| *asb11* | *ASB11* | 8.89E-03 | -3.37 | NM_214792 |
| *zgc:171951* | *LGALS9* | 1.69E-03 | -3.32 | NM_001102630 |
| *casp8ap2* |  | 1.57E-04 | -3.26 | NM_001014334 |
| *gcga* | *GCG* | 8.25E-03 | -2.79 | NM_001008595 |
| [*armc4*](http://www.ensembl.org/Danio_rerio/geneview?gene=ARMC4) | *ARMC4* | 1.26E-02 | -2.67 | XR_117794 |
| *mettl8* | *METTL8* | 9.81E-03 | -2.54 | NM_001007336 |
| *chrna2b* | *CHRNA2* | 6.90E-03 | -2.53 | XM_692206 |
