# Supplementary material for: Identification of Estrogen Target Genes during Zebrafish Embryonic Development through Transcriptomic Analysis
Source: PLoS One. 2013 Nov 6;8(11):e79020. doi: 10.1371/journal.pone.0079020 (PMC3819264; doi:10.1371/journal.pone.0079020)
Supplement: Table S7 — Common estrogen responsive genes at 1 dpf, 2 dpf, 3 dpf and 4 dpf upon E2 treatment. (DOCX) [file pone.0079020.s015.docx]

Table S7. Common estrogen responsive genes at 1 dpf, 2 dpf, 3 dpf and 4 dpf upon E2 treatment

| Overlapping time points | Common genes | Human homologs of the common genes |
| --- | --- | --- |
| 1, 2, 3 and 4 dpf | *vtg1* |  |
| 1 and 2 dpf | *epd, c2cd2l, ddc, loc556849*  *si:ch211-225b11.1, vtg1* | *DDC* |
| 1 and 3 dpf | *dnm1, lhcgr, loc100005283, si:ch211-12e13.2, si:ch73-138i16.1, zgc:193725, vtg3, epd, vtg1* | *DNM1* |
| 1 and 4 dpf | *pomca, vtg3, vtg1* | *POMC* |
| 2 and 3 dpf | *ankrd9, anxa4, cr352285.1, cx28.9, dnajb5, fabp10a, fkbp5, hmgcs1, klf3, klf9, lcn15, loc559127, lpin1, nr1d2a, pik3r3, pnp4b, pnpla3, rhcga, serpinf2, si:dkey-30c15.2, wu:fc15e02, zgc:112242, amh, cyp19a1b, f13a1a, hpx, rgs2, vtg1, epd* | *ANKRD9, DNAJB5, FKBP5, HMGCS1, KLF3, KLF9, LCN15, NR1D2, PIK3R3, PNPLA3, RHCG, SERPINF2, AMH, CYP19A1, F13A1, HPX,* |
| 2 and 4 dpf | *amh, cyp19a1b, f13a1a, hpx, rgs2, ctsbb, vtg1* | *AMH, CYP19A1, F13A1, HPX, CTSB, UNC13C* |
| 3 and 4 dpf | *bdp1, cbln8, dcakd, eif4e1b, gcga, ins, lipc, loc559783, sult1st3, vtg5, zcchc2, zgc:100868, amh, cyp19a1b, f13a1a, hpx, rgs2, vtg1, vtg3* | *C3, CA4, DCAKD, EIF4E1B, GCG, GREB1, KRT20, LIPC, PFKFB4, RGS2, TRIM29, ZCCHC2, AMH, CYP19A1, F13A1, HPX* |
| 1, 2 and 3 dpf | *epd, vtg1* |  |
| 2, 3 and 4 dpf | *amh, cyp19a1b, f13a1a, hpx, rgs2, vtg1* | *AMH, CYP19A1, F13A1, HPX* |
| 1, 3 and 4 dpf | *vtg3, vtg1* |  |
| 1, 2 and 4 dpf | *vtg1* |  |
