# Supplementary material for: Identification of Estrogen Target Genes during Zebrafish Embryonic Development through Transcriptomic Analysis
Source: PLoS One. 2013 Nov 6;8(11):e79020. doi: 10.1371/journal.pone.0079020 (PMC3819264; doi:10.1371/journal.pone.0079020)
Supplement: Table S8 — GO terms sub-grouped into the metabolic process category (in italics). (DOCX) [file pone.0079020.s016.docx]

Table S8. GO terms sub-grouped into the metabolic process category (in italics)

| **Category** | **1 dpf** | | **2 dpf** | | **3 dpf** | | **4 dpf** | |
| --- | --- | --- | --- | --- | --- | --- | --- | --- |
|  | Percent  (%) | p-value | Percent  (%) | p-value | Percent  (%) | p-value | Percent  (%) | p-value |
| *Metabolic process* | 15.63 | **1.43E-02** | 28.13 | **3.02E-02** | 24.93 | **1.14E-17** | 38.32 | **2.53E-06** |
| Hormone biosynthetic process | 3.13 | **9.91E-03** | 3.13 | **4.38E-02** | 2.71 | **9.28E-08** | 4.67 | **2.63E-04** |
| Cellular nitrogen compound metabolic process | 6.25 | **9.55E-04** | 1.56 | 6.21E-01 | 0.54 | 1.01E-01 | 4.67 | **2.52E-02** |
| RNA metabolic process | 3.13 | **5.42E-02** | 3.13 | 2.02E-01 | 1.36 | 1.23E-01 | 5.61 | **5.42E-04** |
| ATP catabolic process | 1.56 | 4.56E-01 | 3.13 | 3.89E-01 | 1.63 | **7.40E-04** | 5.61 | **2.81E-02** |
| Lipid metabolic process | -- | -- | 7.81 | **2.94E-02** | 5.15 | **2.78E-06** | 9.35 | **7.83E-04** |
| Carbohydrate metabolic process | -- | -- | 3.13 | 5.09E-01 | 4.07 | **3.79E-04** | 10.28 | **1.80E-04** |
| Glucose metabolic process | -- | -- | 1.56 | **7.13E-02** | 1.63 | **1.39E-02** | 4.67 | **4.24E-03** |
| Cellular protein metabolic process | -- | -- | 1.56 | **7.13E-02** | 2.17 | **7.58E-02** | 4.67 | **9.10E-02** |
| Xenobiotic metabolic process | -- | -- | 1.56 | 5.06E-01 | 2.71 | **8.26E-05** | 4.67 | **7.17E-03** |
| Energy reserve metabolic process | -- | -- | 1.56 | 4.05E-01 | 2.44 | **3.97E-05** | 4.67 | **1.96E-03** |

Bold p-values represent statistically significant categories (p<0.05).
