# Supplementary material for: Identification of Estrogen Target Genes during Zebrafish Embryonic Development through Transcriptomic Analysis
Source: PLoS One. 2013 Nov 6;8(11):e79020. doi: 10.1371/journal.pone.0079020 (PMC3819264; doi:10.1371/journal.pone.0079020)
Supplement: Table S9 — GO terms sub-grouped into the transport category (in italics). (DOCX) [file pone.0079020.s017.docx]

Table S9. GO terms sub-grouped into the transport category (in italics)

| **Category*** | **1 dpf** | | **2 dpf** | | **3 dpf** | | **4 dpf** | |
| --- | --- | --- | --- | --- | --- | --- | --- | --- |
|  | Percent  (%) | p-value | Percent  (%) | p-value | Percent  (%) | p-value | Percent  (%) | p-value |
| *Transport* | 17.19 | **5.01E-04** | 23.44 | **2.04E-02** | 18.70 | **2.45E-13** | 28.04 | **9.82E-05** |
| Transmembrane transport | 4.69 | 2.49E-01 | 14.06 | **1.52E-02** | 11.38 | **3.37E-12** | 14.02 | **2.57E-03** |
| Ion transport | 10.94 | **5.82E-04** | 6.25 | 3.98E-01 | 7.32 | **4.41E-06** | 8.41 | 9.82E-02 |
| Sodium ion transport | 1.56 | 2.75E-01 | 1.56 | **4.08E-02** | 3.52 | **4.53E-07** | 1.87 | 3.42E-01 |
| Calcium ion transport | 1.56 | 2.68E-01 | 1.56 | **4.08E-02** | 1.90 | **6.57E-03** | 0.93 | **2.34E-02** |
| Amino acid transport | 3.13 | **1.78E-03** | 3.13 | **4.06E-02** | 1.63 | **5.09E-04** | 1.87 | 1.03E-01 |
| Carbohydrate transport | -- | -- | -- | -- | 1.08 | **8.97E-03** | 1.87 | 1.23E-01 |
| Lipid transport | -- | -- | 3.13 | 2.16E-01 | 0.81 | 2.16E-01 | 2.80 | **3.26E-02** |

Bold p-values represent statistically significant categories (p<0.05).
