# Supplementary material for: Identification of Estrogen Target Genes during Zebrafish Embryonic Development through Transcriptomic Analysis
Source: PLoS One. 2013 Nov 6;8(11):e79020. doi: 10.1371/journal.pone.0079020 (PMC3819264; doi:10.1371/journal.pone.0079020)
Supplement: Table S10 — GO terms sub-grouped into the signaling pathways category (in italics). (DOCX) [file pone.0079020.s018.docx]

Table S10. GO terms sub-grouped into the signaling pathways category (in italics)

| **Category*** | **1 dpf** | | **2 dpf** | | **3 dpf** | | **4 dpf** | |
| --- | --- | --- | --- | --- | --- | --- | --- | --- |
|  | Percent  (%) | p-value | Percent  (%) | p-value | Percent  (%) | p-value | Percent  (%) | p-value |
| *Signal transduction* | 28.13 | **6.69E-05** | 17.19 | **4.02E-02** | 15.72 | **3.62E-04** | 18.69 | **6.83E-03** |
| Synaptic transmission | 9.38 | **9.39E-05** | 4.69 | **4.09E-02** | 2.44 | **2.12E-02** | 1.87 | 3.45E-01 |
| Steroid hormone mediated signaling pathway | -- | **--** | 4.69 | 1.72E-04 | 1.08 | **8.42E-03** | 2.80 | **9.33E-03** |
| Cell-cell signaling | -- | **--** | 4.69 | 1.41E-01 | 2.17 | **5.45E-02** | 4.67 | **1.90E-03** |
| Intracellular protein kinase cascade | 1.56 | 2.30E-01 | 1.56 | 4.40E-01 | 1.63 | **1.95E-03** | 3.74 | **1.73E-02** |
| Activation of MAPK activity | 3.13 | **1.51E-02** | -- | -- | 1.36 | **3.70E-03** | 1.87 | **3.84E-02** |
| Cell surface receptor linked signaling pathway | 4.69 | **1.29E-02** | 1.56 | 7.00E-01 | 1.36 | **8.51E-04** | 0.93 | 8.72E-01 |
| Wnt receptor signaling pathway | 1.56 | **4.68E-02** | 3.13 | **4.27E-02** | 0.81 | 4.43E-01 | 4.67 | **1.08E-02** |
| Toll-like receptor 1 signaling pathway | 3.13 | **9.65E-03** | 4.69 | **6.75E-03** | 1.36 | **8.51E-04** | -- | -- |
| Glutamate signaling pathway | 4.69 | **3.50E-06** | -- | -- | -- | -- | -- | -- |
| Nerve growth factor receptor signaling pathway | 1.56 | 3.74E-01 | 3.13 | 2.77E-01 | 2.98 | **2.39E-05** | 2.80 | 2.59E-01 |

Bold p-values represent statistically significant categories (p<0.05).
