# Supplementary material for: Identification of Estrogen Target Genes during Zebrafish Embryonic Development through Transcriptomic Analysis
Source: PLoS One. 2013 Nov 6;8(11):e79020. doi: 10.1371/journal.pone.0079020 (PMC3819264; doi:10.1371/journal.pone.0079020)
Supplement: Table S11 — GO terms sub-grouped into the multicellular organismal development category (in italics). (DOCX) [file pone.0079020.s019.docx]

Table S11. GO terms sub-grouped into the multicellular organismal development category (in italics)

| **Category*** | **1 dpf** | | **2 dpf** | | **3 dpf** | | **4 dpf** | |
| --- | --- | --- | --- | --- | --- | --- | --- | --- |
|  | Percent  (%) | p-value | Percent  (%) | p-value | Percent  (%) | p-value | Percent  (%) | p-value |
| *Multicellular organismal development* | 7.81 | **1.09E-02** | 4.69 | **1.93E-02** | 4.61 | **2.67E-02** | 3.74 | **4.86E-03** |
| Embryo development | 4.69 | **1.09E-02** | 4.69 | 1.03E-01 | 1.90 | **6.60E-02** | 1.87 | **8.84E-02** |
| Fat cell differentiation | -- | **--** | 4.69 | **1.91E-04** | 1.08 | **2.87E-03** | 1.87 | **1.38E-02** |
| Brain development | 1.56 | 4.01E-01 | 4.69 | **1.22E-02** | 1.08 | **1.77E-03** | 3.74 | **1.83E-05** |
| Liver development | 1.56 | 2.03E-01 | 3.13 | **1.93E-02** | 1.08 | **2.67E-02** | 2.80 | **4.86E-03** |
| Muscle development | -- | **--** | 3.13 | **3.95E-02** | 1.08 | **3.17E-02** | 2.80 | **1.27E-03** |
| Gland development | 1.56 | **4.08E-02** | 1.56 | **4.08E-02** | 0.81 | **1.77E-02** | 3.74 | **1.70E-04** |
| Blood vessel development | -- | -- | 3.13 | **3.54E-02** | 0.54 | 2.39E-01 | 1.87 | **1.71E-02** |
| Kidney development | -- | **--** | 1.56 | 4.05E-01 | 1.08 | **2.91E-02** | 0.93 | 5.88E-01 |

Bold p-values represent statistically significant categories (p<0.05).
