# Supplementary material for: Identification of Estrogen Target Genes during Zebrafish Embryonic Development through Transcriptomic Analysis
Source: PLoS One. 2013 Nov 6;8(11):e79020. doi: 10.1371/journal.pone.0079020 (PMC3819264; doi:10.1371/journal.pone.0079020)
Supplement: Table S12 — GO terms sub-grouped into the response to chemical stimulus category (in italics). (DOCX) [file pone.0079020.s020.docx]

Table S12. GO terms sub-grouped into the response to chemical stimulus category (in italics)

| **Category*** | **1 dpf** | | **2 dpf** | | **3 dpf** | | **4 dpf** | |
| --- | --- | --- | --- | --- | --- | --- | --- | --- |
|  | Percent  (%) | p-value | Percent  (%) | p-value | Percent  (%) | p-value | Percent  (%) | p-value |
| Response to chemical stimulus | -- | -- | 7.81 | **9.46E-05** | 3.52 | **1.12E-06** | 6.54 | **9.02E-06** |
| Response to hormone | -- | -- | 4.69 | **2.62E-02** | 2.44 | **1.59E-04** | 6.54 | **9.60E-05** |
| Response to estrogen stimulus | 1.56 | 1.92E-01 | 1.56 | 2.18E-01 | 2.44 | **2.59E-04** | 4.67 | **8.79E-05** |
| Response to drug | 4.69 | **7.99E-02** | 9.38 | **2.51E-02** | 6.50 | **2.36E-07** | 9.35 | **2.85E-06** |

Bold p-values represent statistically significant categories (p<0.05).
