# Supplementary material for: Identification of Estrogen Target Genes during Zebrafish Embryonic Development through Transcriptomic Analysis
Source: PLoS One. 2013 Nov 6;8(11):e79020. doi: 10.1371/journal.pone.0079020 (PMC3819264; doi:10.1371/journal.pone.0079020)
Supplement: Table S13 — Number of genes enriched by the NIH-DAVID tissue enrichment platform. (DOCX) [file pone.0079020.s021.docx]

Table S13. Number of genes enriched by the NIH-DAVID tissue enrichment platform

| Time point | 1 dpf | | 2 dpf | | 3 dpf | | 4 dpf | |
| --- | --- | --- | --- | --- | --- | --- | --- | --- |
|  | No. | % | No. | % | No. | % | No. | % |
| Input genes | 135 | 100 | 103 | 100 | 575 | 100 | 203 | 100 |
| NIH David identified genes | 100 | 74.07 | 68 | 71.92 | 397 | 69.04 | 144 | 70.94 |
| Enriched genes in ZFIN anatomy category | 43 | 43 | 38 | 55.88 | 178 | 44.84 | 64 | 44.44 |
